# Cyclic Nucleotide Gated Channels 7 and 8 Are Essential for Male Reproductive Fertility

**DOI:** 10.1371/journal.pone.0055277

**Published:** 2013-02-12

**Authors:** Meral Tunc-Ozdemir, Claudia Rato, Elizabeth Brown, Stephanie Rogers, Amanda Mooneyham, Sabine Frietsch, Candace T. Myers, Lisbeth Rosager Poulsen, Rui Malhó, Jeffrey F. Harper

**Affiliations:** 1 Department of Biochemistry, University of Nevada, Reno, Nevada, United States of America; 2 Universidade de Lisboa, Faculdade de Ciências de Lisboa, BioFIG, Lisboa, Portugal; 3 Department of Plant Biology and Biotechnology, Centre for Membrane Pumps in Cells and Disease (PUMPKIN), University of Copenhagen, Danish National Research Foundation, Frederiksberg, Denmark; National Taiwan University, Taiwan

## Abstract

The *Arabidopsis thaliana* genome contains 20 CNGCs, which are proposed to encode cyclic nucleotide gated, non-selective, Ca^2+^-permeable ion channels. CNGC7 and CNGC8 are the two most similar with 74% protein sequence identity, and both genes are preferentially expressed in pollen. Two independent loss-of-function *T-DNA* insertions were identified for both genes and used to generate plant lines in which only one of the two alleles was segregating (e.g., *cngc7-1+/−/cngc8-2−/−* and *cngc7-3−/−/cngc8-1*+/−). While normal pollen transmission was observed for single gene mutations, pollen harboring mutations in both *cngc7* and *8* were found to be male sterile (transmission efficiency reduced by more than 3000-fold). Pollen grains harboring *T-DNA* disruptions of both *cngc7* and *8* displayed a high frequency of bursting when germinated *in vitro.* The male sterile defect could be rescued through pollen expression of a *CNGC7* or *8* transgene including a CNGC7 with an N-terminal GFP-tag. However, rescue efficiencies were reduced ∼10-fold when the CNGC7 or 8 included an F to W substitution (F589W and F624W, respectively) at the junction between the putative cyclic nucleotide binding-site and the calmodulin binding-site, identifying this junction as important for proper functioning of a plant CNGC. Using confocal microscopy, GFP-CNGC7 was found to preferentially localize to the plasma membrane at the flanks of the growing tip. Together these results indicate that CNGC7 and 8 are at least partially redundant and provide an essential function at the initiation of pollen tube tip growth.

## Introduction

Fertilization in flowering plants requires a series of carefully coordinated events, including pollen grain germination, pollen tube growth, and directional changes in pollen tube tip growth that guide pollen tubes into the micropyle of an ovule [1]–[3]. When pollen tubes reach a synergid, they burst and discharge sperm cells [4]–[7]. These series of events involve signaling processes that coordinate dynamic changes in the cytoskeleton, ion homeostasis, and membrane trafficking.

Ca^2+^ signals are thought to play a central role in pollen tube tip growth and fertilization [8]–[12]. Evidence from pharmacological and genetic approaches support an important role for at least two different types of Ca^2+^-permeable channels, cyclic nucleotide gated channels (CNGCs) and glutamate receptor-like proteins (GLRs) [13]–[18]. In addition, a knockout of a plasma membrane Ca^2+^-pump ACA9 results in pollen defects that include slow tube growth and a reduced ability to discharge sperm cells to synergids [19]. A double knockout of two pollen-expressed Ca^2+^-dependent protein kinases *CPKs 17* and *34* results in tubes that are slow, short and impaired in their ability to find ovules [20]. Moreover, Ca^2+^ signals have been implicated in regulating the dynamics of the actin cytoskeleton [21], [22] and the activity of Rops, which are small GTPases that can regulate cytoskeletal and secretory processes [22]–[24].

In *Arabidopsis thaliana*, 6 of the 20 CNGCs show detectable expression in pollen [25], [26] and *CNGC18* was shown to be essential for pollen tube tip growth [13], [14]. This is consistent with pharmacological evidence that cyclic nucleotide monophosphate (cNMP) signals can trigger growth-altering Ca^2+^ signals [27]–[29]. While it is possible that cNMP triggered Ca^2+^ signals are a direct result of Ca^2+^ conductance through a CNGC, these channels are also permeable to K^+^, and could be functioning in a way that indirectly triggers a Ca^2+^ release from an internal store [9], [30], [31]. Regardless, a GFP-tagged CNGC18 was found to localize to the growing apical region [13], [14], supporting a model in which cNMP signals have a specific role in regulating signaling and tip growth.

Here we show that two additional pollen-expressed CNGCs (7 and 8) are essential to pollen tube growth. A double knockout of *CNGC7* and *8* results in pollen grains that burst when germinated *in vitro*. A GFP-tagged CNGC7 was found to localize to the plasma membrane, with the strongest GFP signal at the flanks of the pollen tube tip. This favors a model in which the formation and maintenance of pollen tube tip growth requires multiple CNGCs, including CNGC18 and either CNGC7 or 8.

## Results

### *CNGC7* and *8* have Redundant Functions Required for Pollen Transmission

**Figure 1:**
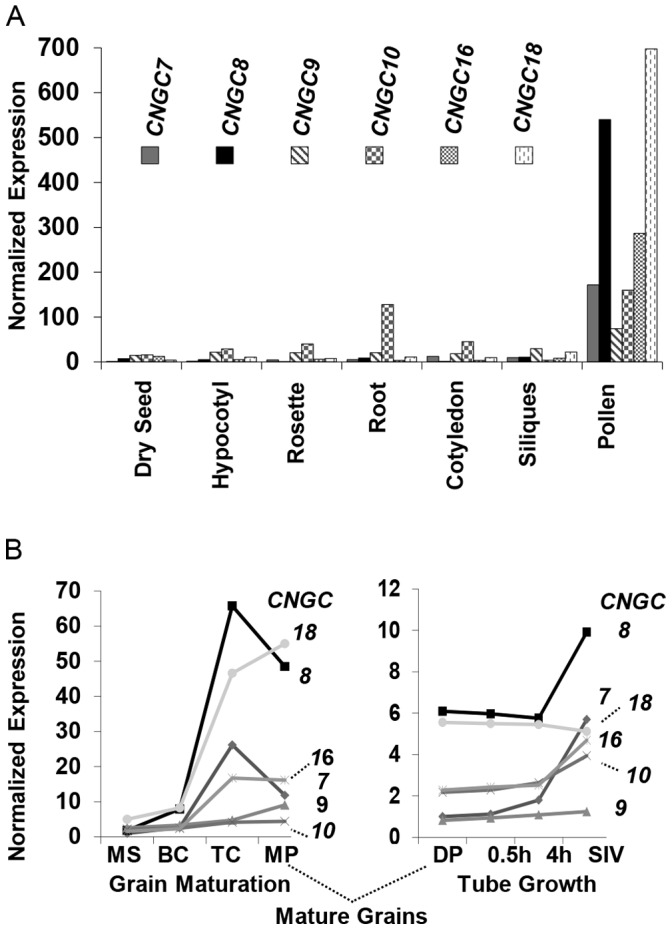
Expression profiles showing preferential pollen expression for six *CNCG*s in *Arabidopsis thaliana*. A) Relative expression levels in different tissues are shown for *CNGC7*, *8*, *9*, *10*, *16*, and *18* (AT1G15990, AT1G19780, AT4G30560, AT1G01340, AT3G48010, and AT5G14870, respectively) obtained from the Arabidopsis eFP Browser (http://bar.utoronto.ca/efp/cgi-bin/efpWeb.cgi) [55] The expression of *CNGC7* in dry seed was arbitrarily set to 1, and the rest of the data normalized accordingly. B**)** Relative expression levels of pollen expressed *CNGC*s at different stages of pollen development obtained from The Pollen Transcriptome Navigator (http://pollen.umd.edu/), which uses data from Honys and Twell, 2004 [25] (left half) and Qin et al., 2009 [26] (right half). Developmental stages are denoted as MS: microspore; BC: bicellular; TC: tricellular; MP: mature pollen; 0.5 h: pollen tube germinated *in vitro* for 30 minutes. 4 h: pollen tube germinated *in vitro* for 4 hours, and SIV: pollen tubes after semi-*in vivo* growth through a stigma. For each data set, the expression of *CNGC7* in microspore and dry pollen were arbitrarily set to 1 and rest of the data normalized accordingly.

**Figure 2:**
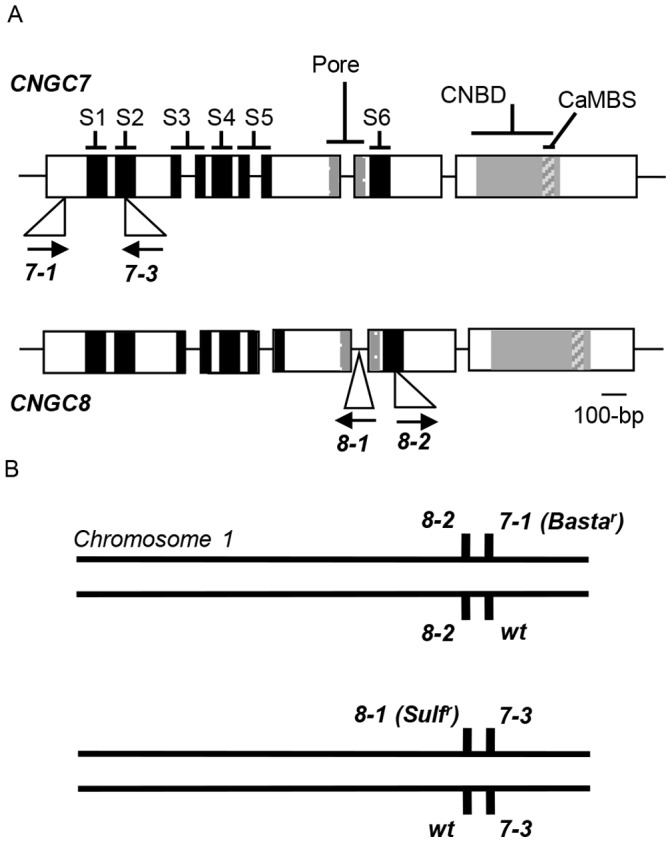
Diagram of the genomic structure of *CNGC7* and *8* and related *T-DNA* insertions. A) Locations of *T-DNA* insertions are shown for *cngc7-1*, *cngc7-3, cngc8-1* and *cngc8-2.* Arrows indicate the direction of the *T-DNA* left border. Coding sequences are highlighted with expanded rectangles; lines indicate introns and flanking DNA sequences. *CNGC7* and *CNGC8* each have five exons, and encode proteins with six transmembrane spanning domains (S1-6; highlighted in black); a pore between S5 and S6 (shaded), a cyclic nucleotide binding domain (CNBD; shaded), and a calmodulin (CaM) binding site (CaMBS; shaded with gray lines) (after Köhler et al., 1999 [55] ). B) A diagram of chromosome 1 showing the arrangement of 2 different combinations of *cngc7* and *8* alleles in which one of the two alleles is segregating with either a *Basta^r^* or *Sulf^r^* marker.

Among the six *CNGC*s that are most highly expressed in *A. thaliana* pollen (Figure 1), *CNGC7* (At1g15990) and *8* (At1g19780) are the two most closely related (74% aa identity). To determine if these genes have redundant functions in pollen development, two independent *T-DNA* gene disruptions for each gene were obtained from publically available *T-DNA* insertion collections: *cngc7-1*, *7-3*, *8-1*, and *8-2* [32]–[34] (Figure 2). The *cngc7-3* and *8-2* alleles have insertions located in exons that encode essential features for a CNGC. As individual mutations, all four insertions showed normal Mendelian segregation when heterozygous plants were self-fertilized or tested for pollen transmission in a manual cross (Table 1).

**Table:** **Table1.** Segregation analysis showing a pollen transmission defect associated with a double knockout of *cngc7/8*.

Cross	F_1_	Segregation of +/− T-DNA		
Female X Male	Total	Expect%^a^	Observed%	p- value^b^
**Crosses with single mutants**				
*cngc7-1*+/−; SELFED	559	75	74.1f, e	0.99
*cngc7-3*+/−; SELFED	178	75	74.7^c^	0.99
*cngc8-1*+/−; SELFED	1347	75	74.5^d^	0.97
*cngc8-2*+/−; SELFED	1409	75	77.4^d^	0.6
*WT* **X** *cngc7-1*+/−	37	50	54^c^	0.95
*WT* **X** *cngc7-3*+/−	71	50	51^c^	0.99
*WT* **X** *cngc8-2*+/−	206	50	50^d^	1
**Crosses with double mutants** (one gene −/−, second gene +/−)				
*cngc7-1*−/−, 8-2+/−; SELFED	637	75	50^d^	<0.0001
*cngc7-1*+/−, 8-2−/−;SELFED	599	75	47.7g,h	<0.0001
*cngc7-3*+/−, 8-1−/−; SELFED	76	75	51c,h	≤0.06
*cngc7-1*−/−, 8-2+/− **X** *WT*	143	50	55^d^	0.7
*cngc7-3*−/−, 8-1+/− **X** *WT*	308	50	55^d^	0.5
*WT* **X** *cngc7-1*+/−, *8-2*−/−	727	50	0^e^	<0.0001
*WT* **X** *cngc7-1*−/−, *8-2*+/−	756	50	0^d^	<0.0001
*WT* **X** *cngc7-3*−/−, *8-1*+/−	5283	50	0^d^	<0.0001

a Expected percentages based on Mendelian segregation.

b Significance determined by the Pearson’s Chi-Squared test with two degrees of freedom.

c PCR genotyping.

d Mutant allele scored by *Sulf* ^r^ marker.

e Mutant allele scored by *Basta^r^* maker.

f 117 by PCR genotyping.

g 313 by PCR genotyping.

h no homozygous double knockout found.

The creation of plants harboring independent sets of double knockouts required the identification of cross-over recombination events between different pairs of *cngc7* and *8 T-DNA* insertions, since *CNGC7* and *8* are closely linked on chromosome 1 (Figure 1). Plant lines with different sets of alleles were allowed to self-fertilize and plant lines with the following 4 genotypes were identified in which only one of the two alleles was segregating: *cngc7-3* (−/−)/*8-1* (+/− *Sulf* ^r^), *cngc7-3* (+/−)/*8-1* (−/−), *cngc7-1* (−/−)/*8-2* (+/− *Sulf* ^r^), and *cngc7-1* (+/− *Basta^r^*)/*8-2* (−/−). For three of these genotype combinations, the segregating allele is linked to a unique selectable marker-gene associated with the *T-DNA* insertion, either providing resistance to glufosinate ammonium (Basta^r^) or sulfadiazine (Sulf^r^).

To try and identify a homozygous *cngc7/8* double knockout, *cngc7/8* combinations segregating only one of the mutant alleles were allowed to self-fertilize, and the progeny was genotyped by PCR assays. In more than 389 progeny analyzed, no plants were found harboring a double homozygous mutation (Table 1). This segregation distortion was corroborated by analyzing the transmission frequencies of the Basta^r^ or Sulf^r^ markers associated with two different *cngc7/8* knockout combinations (∼ 49% marker transmission observed versus 75% expected, n = 1236).

To determine if the inability to segregate a homozygous *cngc7/8* mutant was due to a male or female defect, reciprocal crosses were conducted with three of the different allele combinations. For transmission of the *cngc7/8* double mutation through the female, we observed the expected 50% transmission frequency (n = 451, Table 1). In contrast, no male transmission events were ever detected in more than 6766 progeny analyzed, indicating that pollen transmission was reduced by more than 3000-fold.

**Figure 3:**
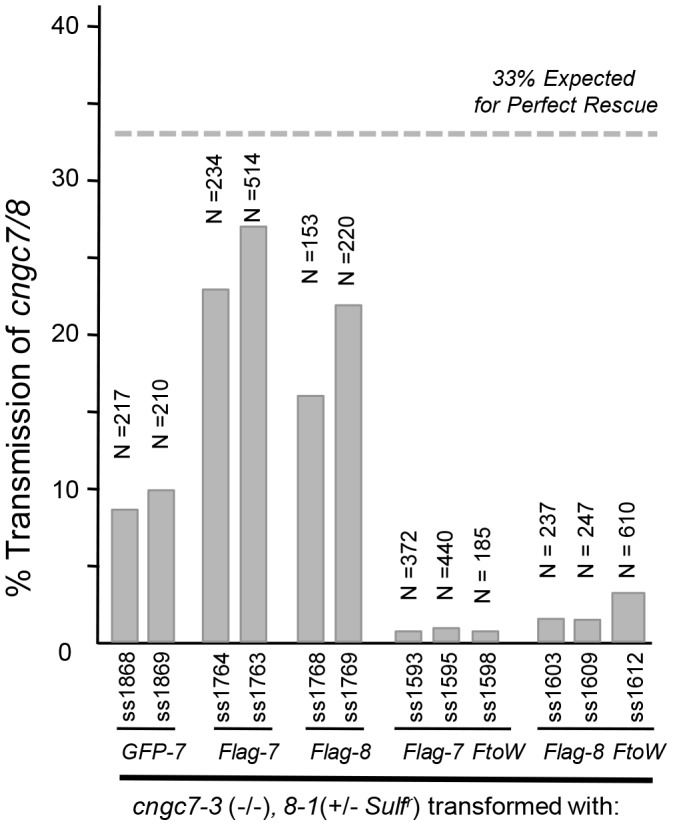
The *cngc7/8* pollen transmission defect can be rescued by *CNGC7* and *8* transgenes. Pollen transmission efficiencies for *cngc7/8* are shown, as scored by the transmission of the *Sulf^r^* marker to F1 progeny. The *Sulf ^r^* marker was associated with the *cngc8-1* allele that was segregating in the parental line. The *cngc7-3* allele was homozygous. Pollen outcrosses were made by manually pollinating females that were wild type or *cngc7-3*(−/−) with equivalent results. All pollen outcrosses were done using male parents that were verified by reciprocal crosses to be hemizygous for the transgene. In these pollen outcross assays, a perfect rescue would result in 33% of the progeny carrying the *Sulf^r^* marker; because only 3 of the 4 meiotic products have the potential to show transmission to F1 progeny (see text). Numbers preceded by ss (seed stock) under each bar represent independent transgenic lines used for outcrossing. Homozygous double knockout seed stocks, created by selfing or out-crossing, are identified in Figure S4. Lines shown displayed typical rescue efficiencies mediated by transgene constructs for *GFP-7 (9p-i-GFP-CNGC7*), *FLAG-7* or *8* (*18p-i-FLAG-CNGC7 or 8*), *FLAG-7* or *8 F to W* (*18p-i-FLAG-CNGC7-F589W* and *18p-i-FLAG-CNGC8-F624W*). Three additional homozyogus rescued lines were obtained (not shown) using a transgene construct ps1687 *18-i-GFP-CNGC8* (ss1402, ss1404, ss1405).

To corroborate that the *cngc7/8* mutations used here represent loss of function null alleles (i.e., knockout), we tested whether the pollen transmission phenotype could be rescued by pollen expression of a transgene encoding either CNGC7 or 8. The N-terminal ends of CNGC7 and 8 were engineered with either GFP or a FLAG-tag, and the transgenes were expressed under the control of either a strong or weak pollen promoter (derived from the regulatory regions upstream of the pollen-expressed Ca^2+^-pump *ACA9* [19] or *CNGC18* [13], [14], respectively). Outcrosses to a female *cngc7-3* (−/−) were done using pollen from plants that were *cngc7-3* (−/−)/*8-1* (+/− *Sulf^r^*) and hemizygous for a transgene encoding either a GFP- or FLAG-tagged CNGC7 or 8. In this situation, meiosis produces pollen with the following 4 genotypes: *cngc7/8* (+/− the *transgene*) and *cngc7/CNGC8* (+/− the *transgene*). Since a *cngc7/8* pollen without a transgene fails to show any transmission (see Table 1), only 3 of the 4 meiotic products have the potential for transmission. Thus, a transgene providing a perfect rescue of *cngc7/8* pollen would result in 33% of the progeny showing the transmission of the *cngc7/8* double knockout, as scored by the segregation of the *Sulf^r^* marker associated with the *cngc8-1* allele. While all transgene variations tested were able to rescue the *cngc7/8* pollen transmission defect to some extent, the best transmission frequencies (23 to 27%) were observed for pollen harboring a *FLAG-CNGC7* transgene expressed under the control of the relatively weak *CNGC18* promoter (Figure 3). These results indicate that the *cngc7/8* mutations studied here result in loss of function phenotypes that can rescued by a transgene encoding either a CNGC7 for CNGC8.

### A Regulatory Site Mutation Impairs the Function of CNGC7 and 8

To generate a mutant plant with only a partial rescue of *cngc7/8*, rescue constructs were engineered to encode mutant versions of CNGC7 and 8 that contained an F589W or F624W substitution, respectively. These substitutions are positioned at a site conserved in plant CNGCs near the carboxyl end of the predicted cyclic nucleotide binding domain (CNBD) and the beginning of a potentially overlapping calmodulin binding-site (CaMBS) (Figure S1).

The respective rescue constructs harboring F to W substitutions were introduced into plants in which the *cngc7* allele was homozygous and the *8* allele was segregating (i.e., *cngc7-3* (−/−)/*8-1* (+/− *Sulf^r^*)). Pollen was then outcrossed and the transmission frequency of a *cngc7/8* double knockout scored in progeny by either PCR genotyping or the expression of a Sulf^r^ phenotype. In contrast to a robust rescue using a wild type version of a *FLAG-CNGC7 or 8*, the incorporation of an F to W substitution (at amino acids 589 and 624, respectively) reduced the pollen transmission efficiency by 10 to 20-fold (Figure 3).

To evaluate whether the F to W substitutions would also compromise the seed set potential in a homozygous mutant, homozygous *cngc7-3/8-1* lines rescued with a FLAG-CNGC7-F589W were identified by PCR genotyping. Although individual plants sometimes showed a reduction in seed set compared to wild type controls, this phenotype was not consistently observed. To understand the cause of this variation, three different plants displaying poor seed set were manually fertilized with the plant’s own pollen. In these cases, the manual self-fertilization was able to restore full seed set. This indicates that the variation in seed set is not a defect associated with the female gametophyte. Rather, the variation is either a result of less pollen being delivered to the stigma, and/or a further decrease in pollen fitness due to unknown variations in growth environments or plant health.

### *cncg7/8* Pollen Grains Burst as they Germinate

To determine why *cngc7/8* mutant pollen are sterile, we first conducted a semi- *in vivo* pollen tube growth assay using pollen from a double knockout mutant segregating a *GFP-CNGC7* rescue construct to 50% of the pollen grains. To set up these assays, receptive stigmas were manually pollinated and then cut and transferred to an agar surface for semi-*in vivo* growth. The only tubes observed to grow were those that showed GFP fluorescence, and therefore were rescued by a *GFP-CNGC7* transgene (n = 27). The absence of any tubes without a GFP-CNGC7 suggested that non-rescued mutant tubes were defective at some early stage of pollen grain germination or tube growth.

**Figure 4:**
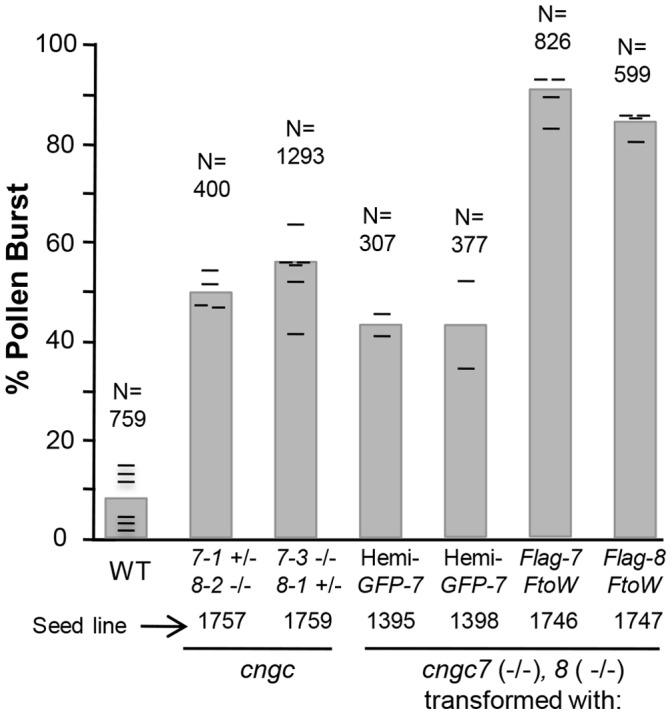
Mutant *cngc7/8* pollen grains burst during *in vitro* germination. Pollen grains were germinated *in vitro* and scored for bursting at the time of germination. A ∼5-fold higher bursting frequency was observed for two different combinations of *cngc7/8* alleles (*cngc7-1+/−, 8-2−/− and cngc7-3−/−, 8-1+/−*) in which only one of the alleles was segregating. An equivalent bursting frequency was observed for a *cngc7-3/8-1* double knockout in which only 50% of the pollen harbored a rescue construct encoding GFP-CNGC7 (GFP-7) (denoted by “Hemi-”). A ∼90% bursting frequency was observed when a *cngc7/8* double knockout was rescued with a FLAG-CNGC7 or 8 harboring an F to W substitution. In these “F to W” examples, the pollen came from a mixed pool of parental plants that were either hemizygous or homozygous for the transgene (*i.e*., at least 50% of the pollen had a “rescue” construct). Each hash mark indicates the % bursting for each of the six independent experiments. Results using two different media (standard, and 10% PEG media) showed equivalent results (n = 3 experiments each).

**Figure 5:**
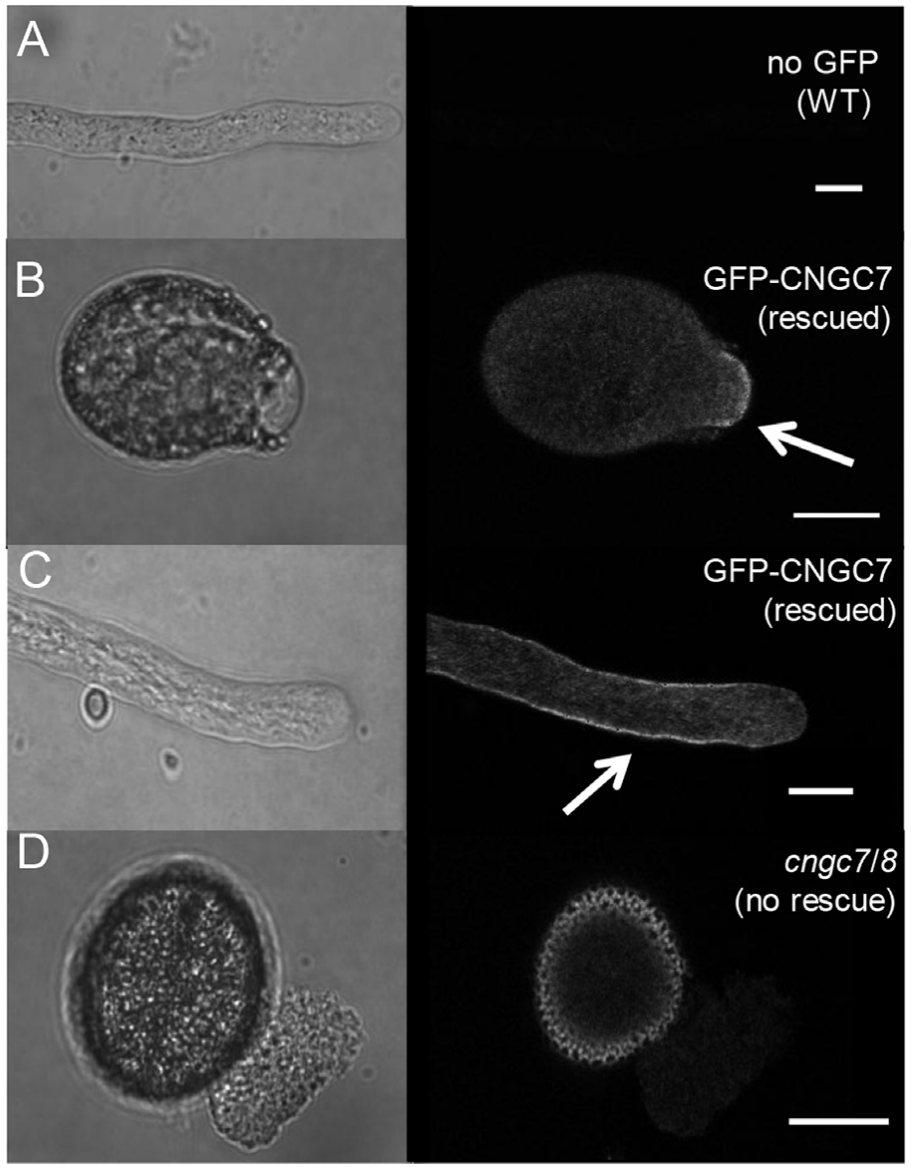
Confocal microscopy showing PM localization for GFP-CNGC7, and the *cngc7/8* bursting defect. Pollen were germinated *in vitro* and imaged. DIC images are shown to the left, and corresponding confocal fluorescence micrographs to the right. A) A negative control showing a wild type pollen tube without any GFP. B and C), GFP-CNGC7 in *cngc7-3−/−, 8-1*−/− showing a tip focused PM (plasma membrane) localization at the emerging tube (B) and the tip shank during tube extension (C). D) A non-rescued pollen from *cngc7-3−/−, 8-1−/−* (segregating a GFP-CNGC7) showing a typical bursting event at germination. Scale bar = 10 µm.

*In vitro* pollen germination assays were then used to specifically evaluate potential defects at early stages of tip growth initiation. In these assays, we evaluated two different combinations of *cngc7/8* alleles in which only one of the alleles was segregating. For both allele combinations, we observed a high frequency (50 to 60%) of pollen grains bursting (Figure 4). In contrast, wild type controls showed an average bursting frequency of less than 10%. For *cngc7/8* mutants, the bursting events usually occurred before any tube growth could be detected (see Figure 5D for example). Similar bursting phenotypes and frequencies were observed using two different standard germination media.

A *cngc7/8* -dependent bursting phenotype was confirmed in two ways (Figure 4). First, *in vitro* germination assays were done with homozygous *cngc7/8* mutants in which 50% of the pollen were expressing a rescue construct encoding GFP-CNGC7 (i.e., parent plants were hemizygous for the transgene). Plants segregating 50% of their pollen with a rescue construct were identified by imaging pollen from each plant for the expression of GFP. Using pollen from these plants, the bursting frequency was near 50% (n = 684). This is consistent with the expectation that 50% of the mutant pollen would be rescued from bursting through the expression of a GFP-CNGC7. This was corroborated by confocal fluorescence microscopy, which revealed that the only tubes to grow beyond the budding stage were those that showed GFP fluorescence (n >50). A second approach was to examine the frequency of bursting in mutant pollen grains from *cngc7/8* plants that harbor transgenes that conferred only a partial rescue. For these pollen expressing either CNGC7-F589W or CNGC8-F624W, the bursting frequency was around 90%, which was about 10-fold higher than wild type controls.

### CNGC7 is Localized to the Plasma Membrane of Pollen Tubes

To provide evidence for the subcellular location of CNGC7, fluorescence confocal microscopy was used to image GFP-CNGC7 in pollen. All imaging was done with homozygous *cngc7/8* mutants that had been rescued by pollen expression of a GFP-CNGC7. Two different promoters were used to drive *GFP-CNGC7* expression. We failed to see detectable levels of GFP using a weak promoter from *CNGC18*, although this promoter was capable of providing low levels of expression sufficient for functional rescues (see Figure 3). Therefore, to obtain high enough expression levels for imaging, we employed a stronger promoter from *ACA9* [19], which resulted in a range of expression levels, from high to barely detectable. Figure 5 shows representative images of cells that have relatively weak but detectable levels of expression. Pollen with very high levels of expression always showed strong fluorescence throughout the cell, including endomembranes (as also observed in transient expression by [13], [14]). However, since functional rescues were observed with very low expression levels (e.g., provided by the *CNGC18* promoter), we posit that images corresponding to low expression levels are more likely to reflect a normal distribution for a CNGC7, and less likely to be an artifact of over-expression [35]. With the imaging parameters used here, autofluorescence was occasionally seen associated with the cell wall (for example, Figure 5D). However, no other significant background fluorescence was detected within cells. In comparison, pollen expressing relatively low levels of GFP-CNGC7 showed strong fluorescent signals predominately associated with the PM at the bud site (Figure 5B), and in growing tubes, predominately at a region flanking the growing tip (Figure 5C).

## Discussion

Genetic evidence presented here indicates that *CNGC7* and *8* function together to provide at least one redundant activity that is essential for pollen fertility in *Arabidopsis thaliana*. Pollen harboring a *cngc7/8* double knockout failed to show any transmission events in pollen outcrosses yielding more than 6000 progeny (expected frequency = 50%, Table 1).

Three lines of evidence suggest that the primary defect in *cngc7/8* pollen occurs at the initiation of pollen tube tip growth, as shown with *in vitro* pollen growth assays (Figure 4). First, pollen grain bursting was observed for approximately 50% of the pollen assayed from mutant plants segregating 50% of their pollen as a *cngc7/8* double knockout. Second, an equivalent bursting frequency was observed for pollen from a plant homozygous for *cngc7/8* in which only half of the pollen harbored a GFP-tagged CNGC7 rescue construct. Third, a higher bursting frequency near 90% was observed for *cngc7/8* pollen partially rescued by a transgene encoding a CNGC7 or 8 that was functionally compromised by an F to W substitution near the end of the proposed cyclic nucleotide binding domain (F589W or F624W, respectively). These *in vitro* results are consistent with the failure to observe tube growth for a *cngc7/8* mutant in a semi-*in vivo* growth assay in which pollen was allowed to germinate on a stigma surface.

Of more than 50 mutations identified with defects associated with pollen germination, only two others are well characterized with an increased bursting frequency, *vgd1* and *anx1/anx2* [6], [7], [21]. *AtAnx1* and *2* encode receptor-like kinases preferentially expressed in pollen, and are proposed to function redundantly in a signaling pathway that controls the timing of pollen tip bursting and sperm discharge when pollen tubes reach the synergid [7]. *AtVGD1* (Vanguard1) encodes a pectin methyltransferase that is important for modifying the pollen cell wall to increase its rigidity [6]. In the absence of a rigid wall, pollen tubes, which have turgor pressure, are more likely to burst.

### A *CNGC18-7/8* Regulatory Node for Pollen Tube Tip Growth?

The *cngc7/8* bursting defect also has similarities to the phenotype observed for *cngc18* null mutants [13], [14]. In the case of *cngc18*, mutant pollen produced short kinky tubes that would often terminate by bursting. While some of the *cngc7/8* pollen also germinated with similar projections, the dominant phenotype appeared to be a bursting projection directly from the pollen grain (Figure 5D). Given the similarities in phenotypes, further research is warranted to determine if CNGC18 might form multimeric complexs with CNGC7 and/or 8. In both plants and animals, CNGCs are thought to function as hetero-multimers [36]–[39], Assuming that hetero-multimers do form between CNGC18 and either CNGC7 or 8, a mutation that disrupts one of the subunits (e.g., CNGC18) might create a dysfunctional or destabilized complex.

### Models for CNGC Regulation of Tip Growth

There are at least two reasonable models, not mutually exclusive, to explain the bursting phenotype associated with a dysfunctional CNGC7/8 multimeric complex. First, the channel complex might be essential for an ion homeostasis mechanism that regulates turgor. When the channel complex is dysfunctional, turgor pressure might increase to a bursting point, as the pollen grain cell wall begins to weaken during germination [6], [40], [41]. Since CNGCs are also permeable to K^+^, they might directly contribute a K^+^ transport involved in turgor regulation. Regulation of K^+^ transport has been proposed as a key feature in the mechanism of tube bursting at the time of sperm discharge [5].

In a second model, the CNGC complex might provide a signaling function that helps coordinate growth cycles at the pollen tube tip. For example, a cyclic nucleotide triggered Ca^2+^ signal might function as a “stop signal” to terminate a growth cycle and restrict growth to a manageable rate. In the absence of such a signal, growth processes might become uncoordinated and thereby make pollen tubes or buds highly susceptible to bursting. This speculation is consistent with a model in which Ca^2+^ signals can block signaling pathways, for example, ROP GTPases, which are implicated in promoting tip growth in pollen tubes and root hairs [42]–[45]. Alternatively, uncoordinated growth cycles might disrupt proper cell wall assembly at the growing tip, and give rise to a structurally weak wall, with a bursting phenotype analogous to that seen with the vanguard mutant [6].

While additional insights will be required to distinguish between these models, evidence here supports a model in which CNGCs 7 and 8 have redundant functions that are essential for the initiation or maintenance of pollen tube tip growth. It remains to be determined as to whether CNGC7 and 8 can form functional interactions with the other four pollen-expressed CNGCs in *A. thaliana*. Regardless, loss of function mutations for *CNGC18 and 7/8* identify at least one CNGC activity that has evolved to be essential to the life cycle of a flowering plant.

## Materials and Methods

Metadata for *CNGC7* and *CNGC8* can be found at TAIR, The Arabidopsis Information Resource (http://www.arabidopsis.org/), under the following accession numbers: At1g15990 and At1g19780, respectively.

### Plant Growth Conditions

*Arabidopsis thaliana* ecotype Columbia (wild type Col-0 and transgenic plants) were germinated on half-strength MS medium (Murashige and Skoog, 1962) with 0.05% (w/v) MES, 0.5% (w/v) sucrose, pH 5.7, and 1% (w/v) agar, under a 24-h light regime, at 21°C. MS medium was supplemented, when necessary, with the appropriate selection marker. Concentrations were as follows: 25 µg/ml hygromycin; 10 µg/ml basta (glufosinate ammonium); 50 µg/ml kanamycin; and 75 µg/ml sulfadiazine.

10-days old seedlings were transplanted to Metro-Mix 200 Series soil (Hummert), fertilized with Triple Ten 10-10-10 containing 40% slow release nitrogen (Growth Products) and grown under a 16-h light/8-h dark regime, at 21°C. All experiments were conducted by comparison of wild type and mutant plants grown side-by-side.

### Isolation of *cngc7* and *cngc8 T-DNA* Insertions

*T-DNA* insertions were identified using the SIGnAL “T-DNA Express” Arabidopsis Gene Mapping Tool (http://signal.salk.edu/cgi-bin/tdnaexpress). *CNGC7 T-DNA* insertion lines were obtained from Syngenta Arabidopsis Insertion Library collection (*cngc7-1*, SAIL_59_F03, harboring a glufosinate-resistance gene, *Basta* ^r^; [32]), and SALK collection (*cngc7-3*, Salk_060871 [33]). *T-DNA* insertion lines for *cngc8* were obtained from GABI-Kat collection (*cngc8-1*, GABI_101C03; *cngc8-2*, GABI_462B04; [34]), all harboring a sulfadiazine-resistance (Sulf^r^) marker within the T-DNA insertion. The glufosinate-ammonium used for *Basta* ^r^ selection and sulfadiazine used for Sulf^r^ are obtained from Sigma-Aldrich (St. Louis,).

The genotypes of all plant lines were confirmed by PCR analysis of genomic DNA using gene-specific and *T-DNA* left border primers. The presence of a wild type *CNGC7* was diagnosed using gene specific primers 1345a and 1345b (Figure S2). The *cngc7-1* and *7-3* insertion alleles were diagnosed using primers 1345br and 638, and 1345a and 792, respectively. *CNGC8* was diagnosed using gene specific primers 960a and 960b. The *cngc8-1* and *8-2* insertion alleles were diagnosed using primers 960a and 958, and 960b and 958, respectively. *T-DNA* border fragments were amplified and sequenced for each line to verify the site of T-DNA insertion.

### Plasmid Constructs Encoding *CNGC7* and *CNGC8*

Plant expression constructs were made in a modified pGreenII vector system [46], with a kanamycin selection marker for bacteria, and a hygromycin marker for plants. The DNA sequence of each construct is provided as a supplemental file (Figure S3). The 9p promoter corresponds to the upstream regulatory region for calcium pump *ACA9* [19]. The 18p promoter corresponds to the upstream regulatory region of CNGC18 [13], [14]. In each construct, the 5′ UTR contains an intron corresponding to a 5′UTR intron from AHA3 [13], [14], [47]. All *CNGC7* constructs contain a genomic sequence for *CNGC7*, which was PCR amplified from Col-0 genomic DNA using primers 1147a and 1147br (Figure S2). All *CNGC8* constructs were made with a *CNGC8* cDNA, which was amplified from *a* Col-0 pSPORT cDNA library (Invitrogen) using primers 1148a and 1148br (Figure S2). F to W substitutions were engineered by a two-step PCR [48]. All sequences derived from PCR reactions were verified by DNA sequencing.

*9p-i-GFP-CNGC7* (ps1300) encodes a GFP-tagged CNGC7, expressed under the control of a *9p* promoter. *18p-i-FLAG-CNGC 7* (ps1692) encodes a FLAG epitope [49], [50] tagged CNGC7, expressed under the control of the *CNGC18* promoter. *18p-i-FLAG-CNGC7(F589W)* (ps1650) is the same as ps1692, but encodes a CNGC7 with an F589W substitution. *18p-i-FLAG-CNGC8* (ps1687) encodes a FLAG epitope tagged CNGC8, expressed under the control of the *CNGC18* promoter. *18p-i-FLAG-CNGC8(F624W)* (ps1685) is the same as ps1687, but encodes a CNGC8 with an F624W substitution. Representative transgenic plants with these constructs are listed in Figure S4 seed stock table.

### Plant Transformation

Transgenic *Arabidopsis thaliana* plants were generated by floral dipping with *Agrobacterium tumefaciens* strain GV3101 [51]. Transgenic plants were selected on MS medium containing hygromycin.

### Pollen Germination

Pollen from open flowers was germinated on standard medium containing 1% low-melting agarose with 0.01% H_3_BO_3_,1 mM CaCl_2_, 5 mM KCl, 10% sucrose, pH 7.5, as modified from [52]. An alternative medium with 10% PEG (polyethylene glycol 4000) was modified from [53], [54] and contained, 0.01% H_3_BO_3_, 3 mM Ca(NO_3_)_2_, 1 mM MgSO_4_, 1 mM KNO_3_, 10% (w/v) PEG, 10% (w/v) sucrose, pH 7.5 with KOH. To make solid medium with PEG, the liquid medium minus PEG was first solidified with 1% low-melting agarose, and then equilibrated with liquid medium including 10% PEG. To enhance the germination rate, one pistil was placed in proximity of the pollen on the germination medium.

### Image Acquisition

Images of GFP fluorescence were collected on a Olympus confocal system (FluoView FV10-ASW 1.5; Olympus) attached to an Olympus microscope (Inverted IX81) using a 60X objective (N.A. = 1.39) and an argon gas laser for generating a 488-nm excitation line. Emission was detected with band pass between 510 and 530 nm. Differential interference contrast (DIC) images were collected on the same system by using a single transmitted light detector. Images were processed by using FluoView software.

## Supporting Information

Figure S1 **Sequence alignment of cyclic nucleotide binding domains (CNBDs) from CNGCs.**
(TIF)

Figure S2 **Primers used in this study.**
(TIF)

Figure S3 **DNA sequences of plasmid constructs used in this study.**
(PDF)

Figure S4 **Seed stocks used in this study.**
(TIF)
