# Supplementary figures and images for: Cyclic Nucleotide Gated Channels 7 and 8 Are Essential for Male Reproductive Fertility
Source: PLoS One. 2013 Feb 12;8(2):e55277. doi: 10.1371/journal.pone.0055277 (PMC3570425; doi:10.1371/journal.pone.0055277)

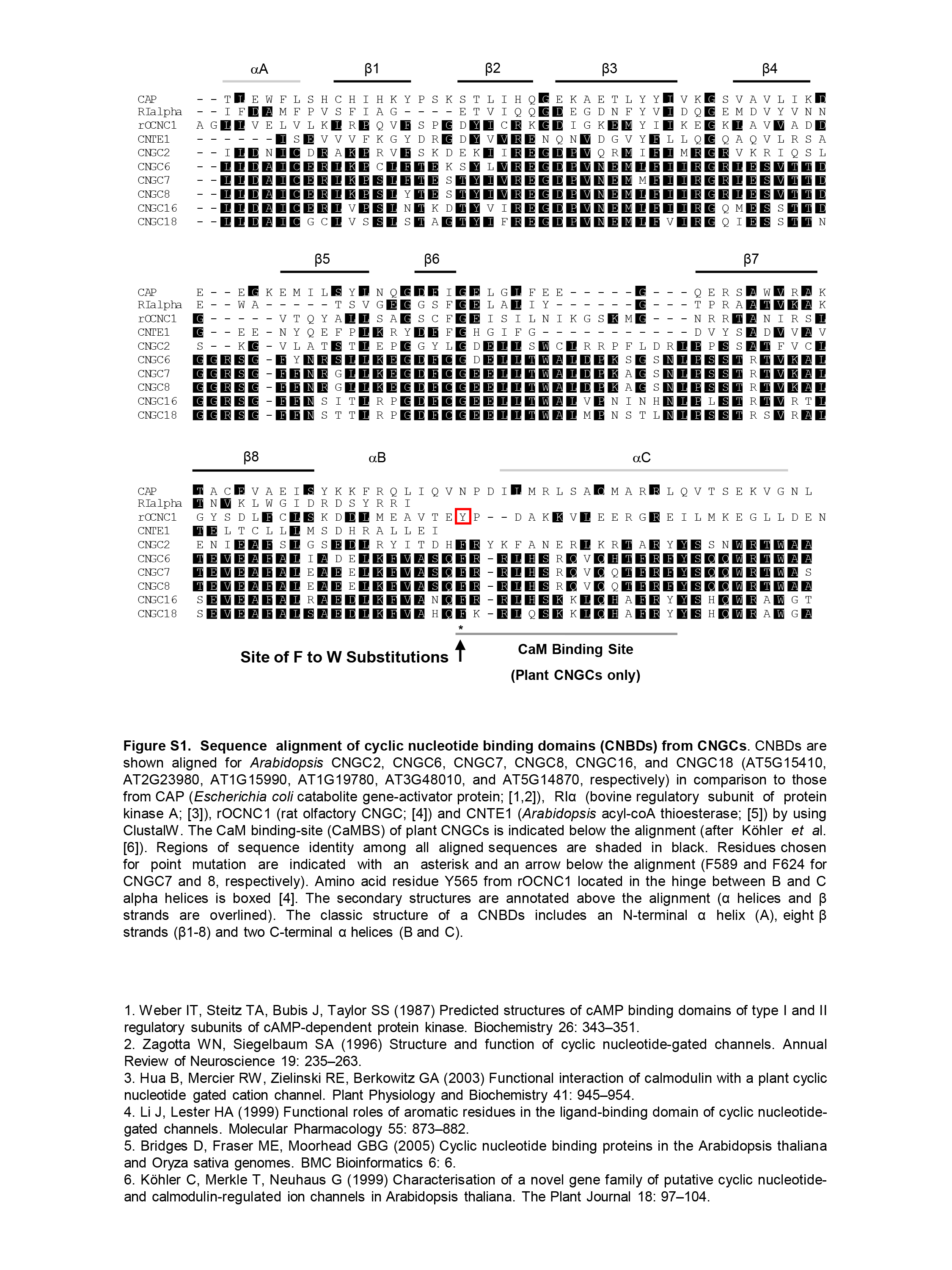

Supplement: Figure S1 — Sequence alignment of cyclic nucleotide binding domains (CNBDs) from CNGCs. (TIF) [file pone.0055277.s001.tif]

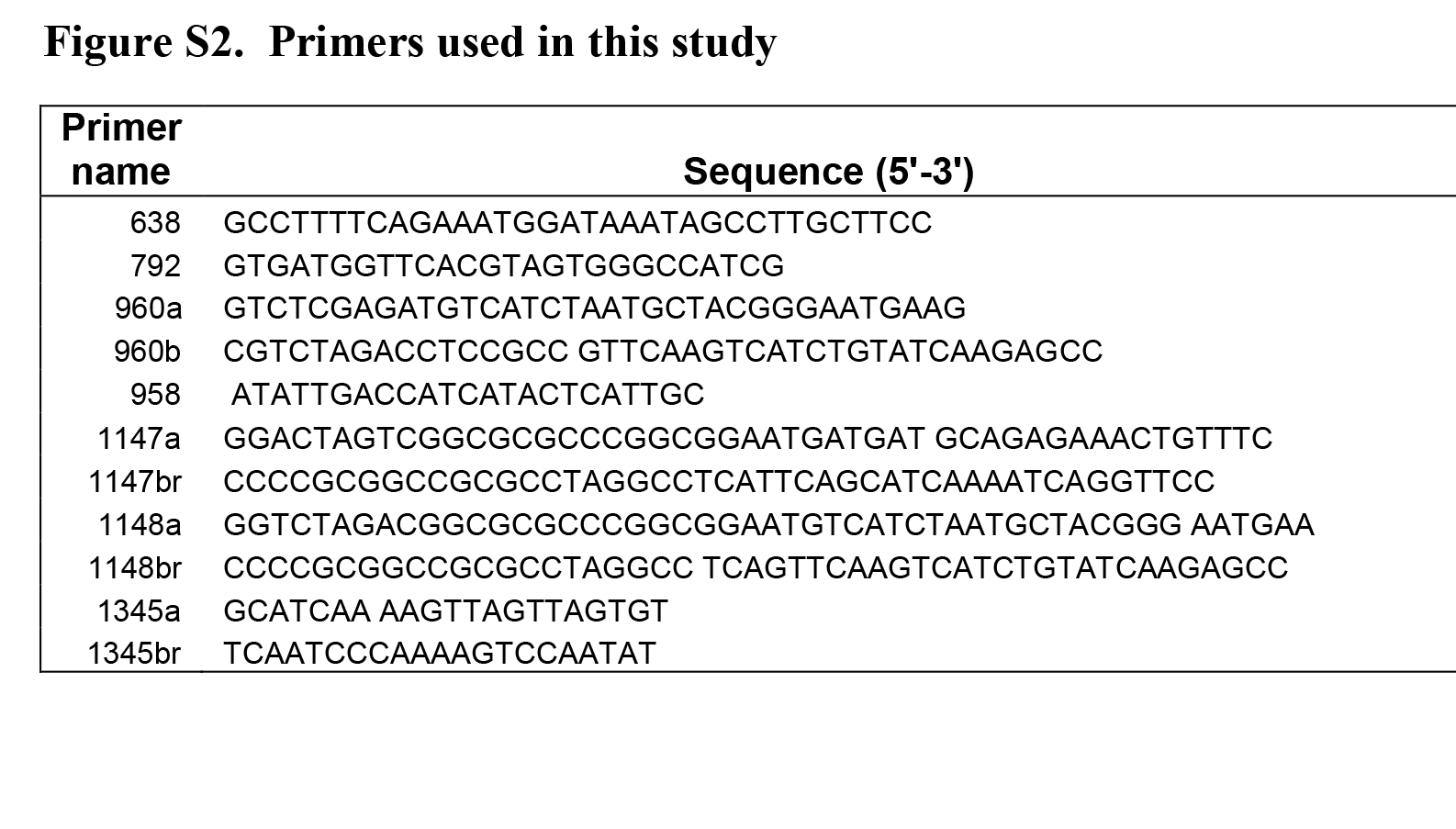

Supplement: Figure S2 — Primers used in this study. (TIF) [file pone.0055277.s002.tif]

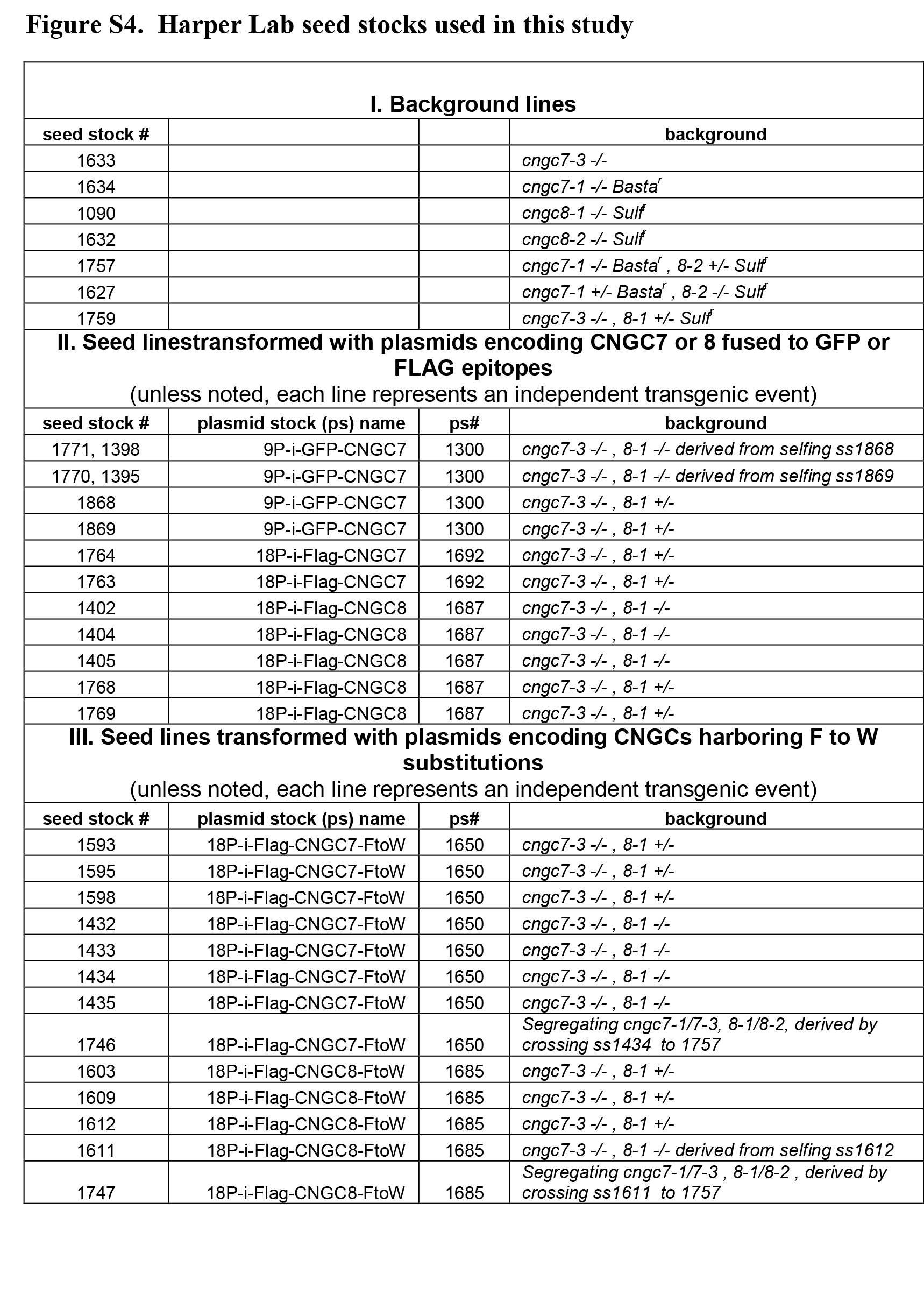

Supplement: Figure S4 — Seed stocks used in this study. (TIF) [file pone.0055277.s004.tif]
