# Supplementary material for: Cyclic Nucleotide Gated Channels 7 and 8 Are Essential for Male Reproductive Fertility
Source: PLoS One. 2013 Feb 12;8(2):e55277. doi: 10.1371/journal.pone.0055277 (PMC3570425; doi:10.1371/journal.pone.0055277)
Supplement: Figure S3 — DNA sequences of plasmid constructs used in this study. (PDF) [file pone.0055277.s003.pdf]

Figure S3. DNA sequences of plasmid constructs used in this study

Plasmid stock 1300, ACA9promoter::intron-GFP-CNGC7(genomic DNA), abbreviated as ps1300, 9p-i-GFP-CNGC7. The *ACA9* promoter with an added intron in the 5' UTR, extends from an SbfI site to SalI, followed by a sequence encoding GFP (green italics). A linker connects GFP to the coding sequence for *CNGC7*, which starts with the first ATG following an AscI site, in the context of GGCGCGCCCGGCGGAATG, and ends with a TGA upstream of a NotI site, in the context of GATTTTGATGCTGAATGA.

TTTTTATCCCCGGAAGCCTGTGGATAGAGGGTAGTTATCCACGTGAAACCGCTAATGCCCCGCAAAGCCTTGATTCACGG
GGCTTTCCGGCCCGCTCCAAAAACTATCCACGTGAAATCGCTAATCAGGGTACGTGAAATCGCTAATCGGAGTACGTGAA
ATCGCTAATAAGGTCACGTGAAATCGCTAATCAAAAAGGCACGTGAGAACGCTAATAGCCCTTTCAGATCAACAGCTTGC
AAACACCCCTCGCTCCGGCAAGTAGTTACAGCAAGTAGTATGTTCAATTAGCTTTTCAATTATGAATATATATATCAATT
ATTGGTCGCCCTTGGCTTGTGGACAATGCGCTACGCGCACCGGCTCCGCCCGTGGACAACCGCAAGCGGTTGCCCACCGT
CGAGCGCCAGCGCCTTTGCCCACAACCCGGCGGCCGGCCGCAACAGATCGTTTTATAAATTTTTTTTTTGAAAAAGAAA
AAGCCCGAAAGGCGGCAACCTCTCGGGCTTCTGGATTTCCGATCCCCGGAATTAGAGATCTTGGCAGGATATATTGTGGT
GTAACGTTATCGATCTGGATTTTAGTACTGGATTTTGGTTTTAGGAATTAGAAATTTTATTGATAGAAGTATTTTACAAA
TACAAATACATACTAAGGGTTTCTTATATGCTCAACACATGAGCGAAACCCTATAAGAACCCTAATTTCCCTTATCGGGA
AACTACTCACACATTATTTATGGAGAAAATAGAGAGAGATAGATTTGTAGAGAGAGACTGGTGATTTCAGCGTACCGAAT
TAATTCTCCCGCGACCAGCCGAGCGAGCTTAGCGAACTGTGGACGAGAACTGTGCCACCAAGCGTAAGGCCGTTCTCTCG
CATTTGCCTTGCTAGGCTCGCGCGAGTTGCTGGCTGAGGCGTTCTCGAAATCAGCTCTTGTTCGGTCGGCATCTACTCTA
TTCCTTTGCCCTCGGACGAGTGCTGGGGCGTCGGTTTCCACTATCGGCGAGTACTTCTACACAGCCATCGGTCCAGACGG
CCGCGCTTCTGCGGGCGATTTGTGTACGCCCGACAGTCCCGGCTCCGGATCGGACGATTGCGTCGCATCGACCCTGCGCC
CAAGCTGCATCATCGAAATTGCCGTCAACCAAGCTCTGATAGAGTTGGTCAAGACCAATGCGGAGCATATACGCCCGGAG
GCGTGGCGATCCTGCAAGCTCCGGATGCCTCCGCTCGAAGTAGCGCGTCTGCTGCTCCATACAAGCCAACCACGGCCTCC
AGAAGAAGATGTTGGCGACCTCGTATTGGGAATCCCCGAACATCGCCTCGCTCCAGTCAATGACCGCTGTTATGCGGCCA
TTGTCCGTCAGGACATTGTTGGAGCCGAAATCCGCGTGCACGAGGTGCCGGACTTCGGGGCAGTCCTCGGCCCAAAGCAT
CAGCTCATCGAGAGCCTGCGCGACGGACGCACTGACGGTGTCGTCCATCACAGTTTGCCAGTGATACACATGGGGATCAG
CAATCGCGCATATGAAATCACGCCATGTAGTGTATTGACCGATTCCTTGCGGTCCGAATGGGCCGAACCCGCTCGTCTGG
CTAAGATCGGCCGCAGCGATCGCATCCATAGCCTCCGCGACCGGCTGAAGAACAGCGGGCAGTTCGGTTTCAGGCAGGTC
TTGCAACGTGACACCCTGTGCACGGCGGGAGATGCAATAGGTCAGGCTCTCGCTGAACTCCCCAATGTCAAGCACTTCCG
GAATCGGGAGCGCGGCCGATGCAAAGTGCCGATAAACATAACGATCTTTGTAGAAACCATCGGCGCAGCTATTTACCCGC
AGGACATATCCACGCCCTCCTACATCGAAGCTGAAAGCACGAGATTCTTCGCCCTCCGAGAGCTGCATCAGGTCGGAGAC
GCTGTCGAACTTTTCGATCAGAAACTTCTCGACAGACGTCGCGGTGAGTTCAGGCTTTTTCATAGGGATCAGCTTGGGCT
GTCCTCTCCAAATGAAATGAACTTCCTTATATAGAGGAAGGGTCTTGCGAAGGATAGTGGGATTGTGCGTCATCCCTTAC
GTCAGTGGAGATGTCACATCAATCCACTTGCTTTGAAGACGTGGTTGGAACGTCTTCTTTTTCCACGATGCTCCTCGTGG
GTGGGGGTCCATCTTTGGGACCACTGTCGGCAGAGGCATCTTGAATGATAGCCTTTCCTTTATCGCAATGATGGCATTTG
TAGGAGCCACCTTCCTTTTCTACTGTCCTTTCGATGAAGTGACAGATAGCTGGGCAATGGAATCCGAGGAGGTTTCCCGA
AATTACCCTTTGTTGAAAAGTCTCAATAGCCCTTTGGTCTTCTGAGACTGTATCTTTGACATTTTTGGAGTAGGGGTACG
ATAACATTAACGTTTACAATTTCGCGCCATTCGCCATTCAGGCTGCGCAACTGTTGGGAAGGGCGATCGGTGCGGGCCTC
TTCGCTATTACGCCAGCTGGCGAAAGGGGGATGTGCTGCAAGGCGATTAAGTTGGGTAACGCCAGGGTTTTCCCAGTCAC
GACGTTGTAAAACGACGGCCAGTGAATTGTAATACGACTCACTATAGGGCGAATTGGGTACCCCTGCAGGAAGCTTGAAC
TGAGAAACCATTGGAAGCAAGAAAGGATCTCAAACAAAAACAGTTGTTATCATTTCATTTGATGATGGAATCGAATCTGA
GTTGTGTTTAGGGTTTCTCTGGGTTAATCTCAGTCTATGTGGGCACATTTTAGTCTTCTACAAATTATAAGACACTCTTC
ACTTATTTAGAAGCTCACTCATCAGAAAAGAAAAAAAAAACTTTGCTCAGTCTCTCATCTGTGTGTTTGATTATATTTTC
AGGCAGAAAACTTTTTTTTGTTCTTGTATTTGATTACAATTAGTTAAATATTTAAGACTTAGTAGAGATAATGCAATCTA
TGTATAAAAATAGAAACAATGATTGGTTATGTCGTGCAACCCAAGTGACCTTAATGTCGTACACACGTTCATCAGGTTAA
AACGAAATTTCTCAGCAACTTCACTATTCTCTCTGTCACATTGTTTTATTCTTTTCCATTATCTTTATTGGAAACTTGAT
TAGAAAGAACTGATTAAGGTATGAAGATAATTAGTGTACCATTCTTCACATGATACGAGGTGAACAATAGTATATCAGAA
TACTAGAATAATTTTATAAGTTATAAAGATGAATGGTATACCACTTATTACAAAACGAAAACATAGTTAGATCTGAGAAG
AAGGAAAGATTTTTGTTTTAAAAAATGAGATGGTTAGATATATGCCATGTTAAGAGATGCCACCATGTTCTTCTTCTTCT
TCTTACCCGGAAAAAACTCTTTCTTCTTCTCTTTCCCATAAAAAGAAACAAAAAAAAACTAACTTCCCACAAATTTTGCC
AAACCAAACCAAAAATAGTAATGATCTCATTCAATTTGAACCCTCTCTCCATTTTCTTTCATAGTTTTTTTTTAGGGTAA
ATCAAAATATTAATGTTGGTTAGAAACTCAAAAATTAGTTTCTCTTTTTTTTTCTCTGTGATTGACAGAGAGGTAAGAGAA
AAAAAAGAGAGAAAGTGTTTCGGTTTTTGTTGCCTTCTCTCGAGTTAGGCCTTCTCTCTTCCGTCACACCATCATTTTTT
TTCTCCATAACGACCAAATCATAAAACCGGTCGTTCCTATTTTTTCTTTCTCTGCGTTTTTTGTTTCGAATTCTTCAATA
GAATTTTAAAAGAAAGAAGAAAGCTGGGTGAGACGAAGGAAGGAAGAGTCGAGCTGCAGTAGTCCACCTTCACTTGCTCT
CTTTTTTCCGAAGAATCTGGTTCGTTTCTCTTTGAATTATCTCTTTCTCTGTTCTTCTTCTTTTTCTAAAGTCTCGAATA
TATCTTCATTATCATTACTTTATTTGCTTCGTTCTTCCGCAGTTTTGTGCAAAGTTTTAACGATTTTTTGGTTTTGAACG
AAGGCACAGAGAGAAGGCGCGTCGACATGGGACTAGTG*ATGGTGAGCAAGGGCGAGGAGCTGTTCACCGGGGTGGTGCCC*
ATCCTGGTCGAGCTGGACGGCGACGTAAACGGCCACAAGTTCAGCGTGTCCGGCGAGGGCGAGGGCGATGCCACCTACGG
CAAGCTGACCCTGAAGTTCATCTGCACCACCGGCAAGCTGCCCGTGCCCTGGCCCACCCTCGTGACCACCTTCACCTACG
GCGTGCAGTGCTTCAGCCGCTACCCCGACCACATGAAGCAGCACGACTTCTTCAAGTCCGCCATGCCCGAAGGCTACGTC
CAGGAGCGCACCATCTTCTTCAAGGACGACGGCAACTACAAGACCCGCGCCGAGGTGAAGTTCGAGGGCGACACCCTGGT
GAACCGCATCGAGCTGAAGGGCATCGACTTCAAGGAGGACGGCAACATCCTGGGGCACAAGCTGGAGTACAACTACAACA
GCCACAACGTCTATATCATGGCCGACAAGCAGAAGAACGGCATCAAGGTGAACTTCAAGATCCGCCACAACATCGAGGAC
GGCAGCGTGCAGCTCGCCGACCACTACCAGCAGAACACCCCCATCGGCGACGGCCCCGTGCTGCTGCCCGACAACCACTA
CCTGAGCACCCAGTCCGCCCTGAGCAAAGACCCCAACGAGAAGCGCGATCACATGGTCCTGCTGGAGTTCGTGACCGCCG
*CCGGGATCACTCACGGCATGGACGAGCTGTACAA*GGGTCTAGAGGGGGGCGCGCCCGGCGGAATGATGATGCAGAGAAAC

TGTTTCGGATTTAACCTAAAGAACCGAGGAGGTGAAAAGAAGAAAGCATCCAAATCTTTCAGGGAAGGAGTTAAAAAAAT
CAGATCAGAAGGTCTAATAACCATCGGTAAATCCGTTACAAGAGCGGTTTTTCCAGAAGACTTAAGAATAACAGAGAAGA
AAATCTTTGATCCTCAAGACAAAACACTTCTCGTATGGAACAGATTGTTCGTCATTTCGTGTATTTTAGCTGTCTCTGTT
GATCCTTTGTTCTTCTATCTTCCCATTGTCGATAATTCCGGAAGCAGCTGTATAGGGATTGATACGAAACTAGCTGTTAC
AACGACGACTCTTAGAACCATTGTTGACGTTTTTTACTTAACTAGAATGGCTCTTCAGTTTCGTACAGCTTATATAGCTC
CTTCTTCTCGTGTGTTTGGTCGTGGTGAGCTTGTGATTGATCCTGCAAAGATCGCTGAACGCTATTTAACCCGTTATTTC
GTCGTCGATTTTCTTGCAGTGCTGCCTTTACCGCAGGTAAAACAGAGTGAAAAGATCCTTTGATGGTTACTAAAAATATT
GGACTTTTGGGATTGATTATGATGTTTTGTGTGGATATTACAGATTGCAGTGTGGAAGTTTCTTCACGGGTCTAAAGGAT
CGGATGTGTTACCAACGAAAACGGCATTGTTGAACATTGTAATTGTGCAGTATATTCCGAGATTCGTGAGGTTTATTCCG
TTAACATCAGAGTTGAAGAAAACTGCAGGAGCTTTTGCCGAAGGAGCTTGGGCTGGTGCTGCTTATTATCTTCTCTGGTA
TATGCTTGCAAGCCACGTAAGCGTTTCTATGTATATATGACTCTTCTTAATTAGTGTTTTGAGACATTTATTCATCATTG
GTCACTTTCTGACTTTACAAACACATTTTGACAGATAACTGGAGCGTTTTGGTACATGTTGTCAGTGGAGCGTAACGATA
CTTGTTGGAGATTTGCGTGTAAAGTTCAGCCAGATCCGCGACTCTGTGTTCAGATTCTGTATTGTGGGACTAAATTTGTG
AGCAGTGGAGAAACTGAATGGATCAAAACAGTCCCTGAGCTTCTTAAGAGCAATTGTTCTGCTAAAGCAGATGATTCAAA
GTTTAACTATGGGATATACGGTCAGGCTATATCTTCAGGCATTGTATCATCAACAACATTTTTCTCCAAGTTCTGTTATT
GTCTATGGTGGGGTCTTCAAAATCTCAGGTAAGGCGGGGGCAACTCATTGTCATTGGTTCAACCGATAGCTTGTGCAGAG
TACTCTTGACATCTTTGGTGTTTTCATGAACAGCACGTTAGGTCAAGGGCTGCAGACAAGTACATTTCCAGGAGAGGTTT
TGTTTTCCATTGCGATTGCTATAGCTGGGCTTCTTCTTTTCGCGCTTCTCATCGGGAATATGCAGACTTATCTTCAGTCA
CTTACTGTTCGTCTCGAGGAAATGAGGATCAAAAGACGTGACTCAGAACAGTGGATGCATCACAGGTCACTTCCACAAAA
TTTGAGGGAAAGAGTCAGACGCTATGATCAATACAAATGGTTAGAGACAAGAGGTGTAGACGAAGAAAACATAGTTCAGA
GTTTACCTAAGGATCTCAGAAGAGACATCAAACGCCATCTCTGTCTGAATTTAGTTAGGAGGGTAAGTTTTCACAATCAG
AATGCTAAAATTGCTTGAGATACTAACTAACTATGTGTTCTGTTTCTACAGGTTCCTCTGTTTGCTAATATGGATGAAAG
ATTACTTGACGCAATCTGTGAGAGATTAAAGCCGAGTCTTTTCACGGAAAGCACTTACATTGTGCGTGAAGGAGACCCCG
TTAACGAAATGATGTTCATAATCCGAGGCCGGTTAGAAAGTGTAACAACAGATGGGGGAAGAAGCGGTTTCTTCAACAGA
GGGTTGTTAAAAGAAGGAGACTTTTGTGGTGAAGAGCTTCTGACATGGGCTCTTGACCCTAAAGCAGGCTCAAACTTACC
TTCTTCCACAAGAACAGTGAAGGCTCTGACTGAAGTAGAGGCTTTCGCTCTTGAAGCTGAAGAGCTGAAGTTTGTAGCAA
GTCAGTTTAGGAGACTTCATAGTCGACAGGTTCAACAAACATTTAGGTTTTACTCTCAGCAATGGAGAACTTGGGCTTCT
TGCTTCATCCAAGCTGCTTGGCGTCGCTATTCAAGAAGGAAAAACGCTGAATTGAGACGGATTGAAGAAAAAGAAGAAGA
ATTGGGTTATGAAGATGAATATGATGATGAGAGTGACAAGAGGCCTATGGTTATCACAAGAAGTGAGTCTTCTTCTAGAC
TGCGTTCAACGATATTTGCATCAAGATTTGCTGCTAATGCATTGAAAGGGCACAGACTTAGGAGCTCAGAGAGTTCAAAG
ACCTTGATAAATCTGCAAAAGCCTCCGGAACCTGATTTTGATGCTGAATGAGGCCTAGGCGCGGTCGCGGGGATCACTAG
TGAATTCGCGGCCGCGGGCCCGGCTGGAGCCACCCGCAGTTCGAAAAATGAGAGCTCGAATTTCCCCGATCGTTCAAACA
TTTGGCAATAAAGTTTCTTAAGATTGAATCCTGTTGCCGGTCTTGCGATGATTATCATATAATTTCTGTTGAATTACGTT
AAGCATGTAATAATTAACATGTAATGCATGACGTTATTTATGAGATGGGTTTTTATGATTAGAGTCCCGCAATTATACAT
TTAATACGCGATAGAAAACAAAATATAGCGCGCAAACTAGGATAAATTATCGCGCGCGGTGTCATCTATGTTACTAGATC
GGGAATTCAGCTTTTGTTCCCTTTAGTGAGGGTTAATTCCGAGCTTGGCGTAATCATGGTCATAGCTGTTTCCTGTGTGA
AATTGTTATCCGCTCACAATTCCACACAACATACGAGCCGGAAGHCATAAAGTGTAAAGCCTGGGGTGCCTAATGAGTGA
GCTAACTCACATTAATTGCGTTGCGCTCACTGCCCGCTTTCCAGTCGGGAAACCTGTCGTGCCAGCTGCATTAATGAATC
GGCCAACGCGCGGGGAGAGGCGGTTTGCGTATTGGGCGCTCTTCCGCTTCCTCGCTCACTGACTCGCTGCGCTCGGTCGT
TCGGCTGCGGCGAGCGGTATCAGCTCACTCAAAGGCGGTAATACGGTTATCCACAGAATCAGGGGATAACGCAGGAAAGA
ACATGAAGGCCTTGACAGGATATATTGGCGGGTAAACTAAGTCGCTGTATGTGTTTGTTTGAGATCTCATGTGAGCAAAA
GGCCAGCAAAAGGCCAGGAACCGTAAAAAGGCCGCGTTGCTGGCGTTTTTCCATAGGCTCCGCCCCCCTGACGAGCATCA
CAAAAATCGACGCTCAAGTCAGAGGTGGCGAAACCCGACAGGACTATAAAGATACCAGGCGTTTCCCCCTGGAAGCTCCC
TCGTGCGCTCTCCTGTTCCGACCCTGCCGCTTACCGGATACCTGTCCGCCTTTCTCCCTTCGGGAAGCGTGGCGCTTTCT
CATAGCTCACGCTGTAGGTATCTCAGTTCGGTGTAGGTCGTTCGCTCCAAGCTGGGCTGTGTGCACGAACCCCCCGTTCA
GCCCGACCGCTGCGCCTTATCCGGTAACTATCGTCTTGAGTCCAACCCGGTAAGACACGACTTATCGCCACTGGCAGCAG
CCACTGGTAACAGGATTAGCAGAGCGAGGTATGTAGGCGGTGCTACAGAGTTCTTGAAGTGGTGGCCTAACTACGGCTAC
ACTAGAAGAACAGTATTTGGTATCTGCGCTCTGCTGAAGCCAGTTACCTTCGGAAGAAGAGTTGGTAGCTCTTGATCCGG
CAAACAAACCACCGCTGGTAGCGGTGGTTTTTTTGTTTGCAAGCAGCAGATTACGCGCAGAAAAAAAGGATCTCAAGAAG
ATCCTTTGATCTTTTCTACGGGGTCTGACGCTCAGTGGAACGAAAACTCACGTTAAGGGATTTTGGTCATGAGATTATCA
AAAAGGATCTTCACCTAGATCCTTTTAAATTAAAAATGAAGTTTTAAATCAATCTAAAGTATATATGTGTAACATTGGTC
TAGTGATTAGAAAAACTCATCGAGCATCAAATGAAACTGCAATTTATTCATATCAGGATTATCAATACCATATTTTTGAA
AAAGCCGTTTCTGTAATGAAGGAGAAAACTCACCGAGGCAGTTCCATAGGATGGCAAGATCCTGGTATCGGTCTGCGATT
CCGACTCGTCCAACATCAATACAACCTATTAATTTCCCCTCGTCAAAAATAAGGTTATCAAGTGAGAAATCACCATGAGT
GACGACTGAATCCGGTGAGAATGGCAAAAGTTTATGCATTTCTTTCCAGACTTGTTCAACAGGCCAGCCATTACGCTCGT
CATCAAAATCACTCGCATCAACCAAACCGTTATTCATTCGTGATTGCGCCTGAGCGAGACGAAATACGCGATCGCTGTTA
AAAGGACAATTACAAACAGGAATCGAATGCAACCGGCGCAGGAACACTGCCAGCGCATCAACAATATTTTCACCTGAATC
AGGATATTCTTCTAATACCTGGAATGCTGTTTTCCCTGGGATCGCAGTGGTGAGTAACCATGCATCATCAGGAGTACGGA
TAAAATGCTTGATGGTCGGAAGAGGCATAAATTCCGTCAGCCAGTTTAGTCTGACCATCTCATCTGTAACAACATTGGCA
ACGCTACCTTTGCCATGTTTCAGAAACAACTCTGGCGCATCGGGCTTCCCATACAATCGGTAGATTGTCGCACCTGATTG
CCCGACATTATCGCGAGCCCATTTATACCCATATAAATCAGCATCCATGTTGGAATTTAATCGCGGCCTTGAGCAAGACG
TTTCCCGTTGAATATGGCTCATAACACCCCTTGTATTACTGTTTATGTAAGCAGACAGTTTTATTGTTCATGATGATATA
TTTTTATCTTGTGCAATGTAACATCAGAGATTTTGAGACACAACGTGGCTTTGTTGAATAAATCGAACTTTTGCTGAGTT
GAAGGATCAGATCACGCATCTTCCCGACAACGCAGACCGTTCCGTGGCAAAGCAAAAGTTCAAAATCACCAACTGGTCCA
CCTACAACAAAGCTCTCATCAACCGTGGCTCCCTCACTTTCTGGCTGGATGATGGGGCGATTCAGGCGATCCCCATCCAA
CAGCCCGCCGTCGAGCGGGCT

Plasmid stock 1650, CNGC18promoter::intron-Flag-CNGC7(F589W)(genomicDNA), abbreviated as ps1650, 18p-i-Flag-CNGC7(F589W). The *CNGC18* promoter with an added intron in the 5′ UTR, extends from an SbfI site to the first SalI site, followed

by a sequence encoding FLAG epitope tag (green italics). A linker connects the Flag tag to the coding sequence for *CNGC7* with an F589 to W substitution, which starts with the first ATG following an AscI site, in the context of GGCGCGCCCGGCGGAATG, and ends with a TGA upstream of a NotI site, in the context of GATTTTGATGCTGAATGA. The F to W substitution with a silent NheI diagnostic site is in the context of GCTAGCCAGTGG

TTTTTATCCCCGGAAGCCTGTGGATAGAGGGTAGTTATCCACGTGAAACCGCTAATGCCCCGCAAAGCCTTGATTCACGGGGCTTTCCG
GCCCGCTCCAAAAACTATCCACGTGAAATCGCTAATCAGGGTACGTGAAATCGCTAATCGGAGTACGTGAAATCGCTAATAAGGTCACG
TGAAATCGCTAATCAAAAAGGCACGTGAGAACGCTAATAGCCCTTTCAGATCAACAGCTTGCAAACACCCCTCGCTCCGGCAAGTAGTT
ACAGCAAGTAGTATGTTCAATTAGCTTTTCAATTATGAATATATATATCAATTATTGGTCGCCCTTGGCTTGTGGACAATGCGCTACGC
GCACCGGCTCCGCCCGTGGACAACCGCAAGCGGTTGCCCACCGTCGAGCGCCAGCGCCTTTGCCCACAACCCGGCGGCCGGCCGCAACA
GATCGTTTTATAAATTTTTTTTTTTGAAAAAGAAAAAGCCCGAAAGGCGGCAACCTCTCGGGCTTCTGGATTTCCGATCCCCGGAATTA
GAGATCTTGGCAGGATATATTGTGGTGTAACGTTATCGATCTGGATTTTAGTACTGGATTTTGGTTTTAGGAATTAGAAATTTTATTGA
TAGAAGTATTTTACAAATACAAATACATACTAAGGGTTTCTTATATGCTCAACACATGAGCGAAACCCTATAAGAACCCTAATTTCCCT
TATCGGGAAACTACTCACACATTATTTATGGAGAAAATAGAGAGAGATAGATTTGTAGAGAGAGACTGGTGATTTCAGCGTACCGAATT
AATTCTCCCGCGACCAGCCGAGCGAGCTTAGCGAACTGTGGACGAGAACTGTGCCACCAAGCGTAAGGCCGTTCTCTCGCATTTGCCTT
GCTAGGCTCGCGCGAGTTGCTGGCTGAGGCGTTCTCGAAATCAGCTCTTGTTCGGTCGGCATCTACTCTATTCCTTTGCCCTCGGACGA
GTGCTGGGGCGTCGGTTTCCACTATCGGCGAGTACTTCTACACAGCCATCGGTCCAGACGGCCGCGCTTCTGCGGGCGATTTGTGTACG
CCCGACAGTCCCGGCTCCGGATCGGACGATTGCGTCGCATCGACCCTGCGCCCAAGCTGCATCATCGAAATTGCCGTCAACCAAGCTCT
GATAGAGTTGGTCAAGACCAATGCGGAGCATATACGCCCGGAGGCGTGGCGATCCTGCAAGCTCCGGATGCCTCCGCTCGAAGTAGCGC
GTCTGCTGCTCCATACAAGCCAACCACGGCCTCCAGAAGAAGATGTTGGCGACCTCGTATTGGGAATCCCCGAACATCGCCTCGCTCCA
GTCAATGACCGCTGTTATGCGGCCATTGTCCGTCAGGACATTGTTGGAGCCGAAATCCGCGTGCACGAGGTGCCGGACTTCGGGGCAGT
CCTCGGCCCAAAGCATCAGCTCATCGAGAGCCTGCGCGACGGACGCACTGACGGTGTCGTCCATCACAGTTTGCCAGTGATACACATGG
GGATCAGCAATCGCGCATATGAAATCACGCCATGTAGTGTATTGACCGATTCCTTGCGGTCCGAATGGGCCGAACCCGCTCGTCTGGCT
AAGATCGGCCGCAGCGATCGCATCCATAGCCTCCGCGACCGGCTGAAGAACAGCGGGCAGTTCGGTTTCAGGCAGGTCTTGCAACGTGA
CACCCTGTGCACGGCGGGAGATGCAATAGGTCAGGCTCTCGCTGAACTCCCCAATGTCAAGCACTTCCGGAATCGGGAGCGCGGCCGAT
GCAAAGTGCCGATAAACATAACGATCTTTGTAGAAACCATCGGCGCAGCTATTTACCCGCAGGACATATCCACGCCCTCCTACATCGAA
GCTGAAAGCACGAGATTCTTCGCCCTCCGAGAGCTGCATCAGGTCGGAGACGCTGTCGAACTTTTCGATCAGAAACTTCTCGACAGACG
TCGCGGTGAGTTCAGGCTTTTTCATAGGGATCAGCTTGGGCTGTCCTCTCCAAATGAAATGAACTTCCTTATATAGAGGAAGGGTCTTG
CGAAGGATAGTGGGATTGTGCGTCATCCCTTACGTCAGTGGAGATGTCACATCAATCCACTTGCTTTGAAGACGTGGTTGGAACGTCTT
CTTTTTCCACGATGCTCCTCGTGGGTGGGGGTCCATCTTTGGGACCACTGTCGGCAGAGGCATCTTGAATGATAGCCTTTCCTTTATCG
CAATGATGGCATTTGTAGGAGCCACCTTCCTTTTCTACTGTCCTTTCGATGAAGTGACAGATAGCTGGGCAATGGAATCCGAGGAGGTT
TCCCGAAATTACCCTTTGTTGAAAAGTCTCAATAGCCCTTTGGTCTTCTGAGACTGTATCTTTGACATTTTTGGAGTAGGGGTACGATA
ACATTAACGTTTACAATTTCGCGCCATTCGCCATTCAGGCTGCGCAACTGTTGGGAAGGGCGATCGGTGCGGGCCTCTTCGCTATTACG
CCAGCTGGCGAAAGGGGGATGTGCTGCAAGGCGATTAAGTTGGGTAACGCCAGGGTTTTCCCAGTCACGACGTTGTAAAACGACGGCCA
GTGAATTGTAATACGACTCACTATAGGGCGAATTGGGTACCGTTTAAACAAGCTTCCTAGCCCTGCAGGGGATCCTGGGTTTTGTATCT
AAAGATCAAAGGAATAACATTTTTGGGAGATGAGAAACTTCAAAAGATGCGATTTAGTTTTTGTGGAAAAGTGGGGAATTTAATTCGAT
TGACGTTAGGAACTTTTTCTCTATGGATTCTTTGGGTTTGTGATTAATGGCATTGAAGGTACAAAGGAAAAGGTTTGGCCAACAAAATC
AAGAACCACAAAGAGAGAGAGAGAGAGGAAACACACAGACAAAACATATGAAAGAATGATTAAGATAACTGCCACAAATATAACTGCAA
AATAACATACTCCTTAAGAAAATTAGTGGGGCCTTCTTTGTTATGGAAGAATGTGCAAATCTTTTTATATGGTTAAGTATATTAGTATT
ATTATATCAAAATTCATTTTTGACTATATATAGGTGATTGTGTCGAAATTCACGTAAGATCTTTGTAACATTTTTTCACACTAGTTAAT
TTTACATGTATTATAGATTCGCTGCAAAAAAAAAAAAAAAAAAAAAAAAAGAAAATAGAAAAAATTTCTGTTTTTTCACACTAGGCCGATTT
GATTGATCAAAACCGATTTTAAAAGCTTAAACTAATAAATCGATTTCATTCACCAATTTATTAAAATTAGATCGTTTTGGAGGTTCAAG
AATATCTGGTCGCTGTTTAAGACGATATTTTGAATCTTGAACCGTTGAAACTATATATACTAGGTATATTTACTGTATGTTGTGAAAGG
GTTTACTATCACATGGCTATTATATAAGACCGTACTAACATATGCAAATATTATATGTGGATCAAAGTACCACAAATTTTGAGTGAACC
AAAAACAAAAGGTTAGAGATAGAAAAATATGCATGTAAAAAATAATAAGAGATGAAAGAAATATTAATGGGAGATGGGTGGGAAATGAA
TAGATTGGTGTCTTCGTCACTTTTACTTTTTTTTTTTTTTTATTGTTTGTTTGATAGTACCAAGAGATTCTCTATTCTATTCTTTCGTGA
TGGTTTATTTTAGGGTATATTACGTGACCTGTAGTTCATCCTCTCCAACTCTCAAACCCACAAATTATTCACTATACATATATCCAAAG
ATTATTTATTCAAAGATATGACTAAAAATCTCTATACACGTGTGTATGTAATAGAAAGGTGCAAGGATTTTGGTGTGGCACCACCTTCA
AGACTTTTTTTTTATCAAGACCTAACTCTTACCACTTTGAAAGCAGCTTATCTTATTTCTTTAGATAAAACAATTGATAAGCGAGTGCT
TTAAACTTCACAAAACTACAATCTACTTTTTATTCGATAAAACAAAAGATTTTATTAGTCTTTTTCATTAGGGTCCCAAATGATTAATA
TGTTCACGATCACGAAATTTCTTCTTGCGTCCCGTAAGCATTTATATTAATCAAACAAAAAGGAAATTTGTTGCTTCCTACAATGGGAA
GTGGGAACCTTTGCCATCTTCATAAATGTTTTAAATCTTTTTTTTTTCTTTCTAATGTGTGTTTCATAAACACCAGCGACTTTATTGAA
AAGAGGAACACGTAAATTGTATTCTCAAAGAACAATTTTTTTAGGAAGAGTAACATTTGTTTTCCTTATCTTAATATAAGCAACGAAAT
CAATCGTGCATATAAAAGTGAAATGCTATTTTGAATTTTCTAAACTTGACTAGCAAATTTTCACTAAAAAATAAAATTAAAAAGTAACG
ATAAAGTAGTTGTCGTATAGTCTACTCCACATGAATAAAATAATAAAGTAAATCAATAGAATGTAAATCTCTTAATTAATCATACTTAT
TATATAATCACAGTGGCAAAGTGGAATAAAAAAAAAAGAGGAGAATTGGATCCCGTCGGATTCTATATTTAAAGATCGCTAAACATCTC
AAATCTGCCACACGTGGTCATACTTAATAGCAGCCATTAACGAAGTAATAAATTAGTCGATAATAATGTTTAACCAACATCGTATTTCT
AGATCCATGAATTAGTTGGAGTAATTAACATCATTGTTTCTTAATTACGTTTAACAAGCATAGAGAAAACAATATTGTCACATATGTTA
GTATAAGTTATCATAATCATTTATAAAAGCTTGTTATTAATTTTTTATATTGAAATTACTACTTTGTGTCTAATCGGAAAATGATGGAG
TACTAAAATTTGAAGGACTAAAATACGTCATTATTGTTAGAGAAAACAATTACGCATAAGTGTGATTTTTTGGAAGTGGAAAAGTTAGT
ATGTCCCTCTATATGATATGATGGAAGTTGTTGTTCTTTTTGTGTGGATAAACATCATTTTTCCGTGACGTAATTTTTAATTTCAGTAT
TCATTTTATTATTCTCACTTCTTATTATCCCTTTTATATTCGCATAATTGTTTCTAAGGATTTCTATTCCCTTTTTGTTATTTTATAGG
TACACTATCTTATGGATGGTGTCAACAAGCATGTACACTATCTTCCCACTACCACATATTGTCACAGAATGTTTGGTACGCGTGCTTCT
ATATACTTTTAATAGTACATAACAACAACAAATGAATTATGTAAGACAATTTGTTAAACTGATGTCAAAAACGGAAATACTACAAAAGA
CAGTATAATTGAATTACGGGTTTAATGTGTTGACAAAATATAATAAATGTAGCTCATAAAACTTCAATAATTCACTTAAATAAATTCTA
ATGAACCTCATAACGAATTAGATATTTTAACTAATCTTATCATGGTTTTACGAATTAATTATAATTTTTTTTTTTTATTCTTTGAACAAT
TGAACTAAACAATATTAAACCGGACTTTAAGTTAAAACTACTAAAAACATTATTAGTTTATCAATAGAAATGATCATAGCAATTGTATA
CATTATAATGTGTAATTTTAAAATCCTAAAGCATATATAGTATATATCCCTTTGATTTTCATAGAAAAAAAGTAGAAAAAAATAACTAT
TGGGCCGATCTATAACTATTGGGCCGGTGGGTCCAACAACTAAGAACAGTAGATATTGTGTCGATTGAAGTTTTGAGATGCAAAACGTG

TGCTGTTTTGAGAACACAAAAACAGCATAAACAAACGAGACCCGCAACTATAACATTCCGTAAATACCCTCATTCTCCCATCATATTTT
CAAAAACTACCCTTCCCCCTATTTCCATGGCCCTTCTCTCAGTTTTCCCGCGAGAAATCCTCTCCTTCTTCCATAATCATCAAAAACAA
AATGCAAAAACCCAAAAAAACAAAAAATCACACGACGCAACCACCTAGCCTCTCTGTCCATCAATCTCAATCTCTCTCTGTGTTAATGC
GGAATCCGATTTAACACCGGAATCCGCCATGGAAGGGAGGCGGTGTCGAGGTGCAGTAGTCCACCTTCACTTGCTCTCTTTTTTCCGAA
GAATCTGGTTCGTTTCTCTTTGAATTATCTCTTTCTCTGTTCTTCTTCTTTTTCTAAAGTCTCGAATATATCTTCATTATCATTACTTT
ATTTGCTTCGTTCTTCCGCAGTTTTGTGCAAAGTTTTAACGATTTTTTGGTTTTGAACGAAGGCACAGAGAGAAGGCGCGTCGAC*ATGG*
*ACTACAAAGACGATGACGACAAG*GTCGACGGCGCGCCCGGCGGAATGATGATGCAGAGAAACTGTTTCGGATTTAACCTAAAGAACCGA
GGAGGTGAAAAGAAGAAAGCATCCAAATCTTTCAGGGAAGGAGTTAAAAAAATCAGATCAGAAGGTCTAATAACCATCGGTAAATCCGT
TACAAGAGCGGTTTTTCCAGAAGACTTAAGAATAACAGAGAAGAAAATCTTTGATCCTCAAGACAAAACACTTCTCGTATGGAACAGAT
TGTTCGTCATTTCGTGTATTTTAGCTGTCTCTGTTGATCCTTTGTTCTTCTATCTTCCCATTGTCGATAATTCCGGAAGCAGCTGTATA
GGGATTGATACGAAACTAGCTGTTACAACGACGACTCTTAGAACCATTGTTGACGTTTTTTACTTAACTAGAATGGCTCTTCAGTTTCG
TACAGCTTATATAGCTCCTTCTTCTCGTGTGTTTGGTCGTGGTGAGCTTGTGATTGATCCTGCAAAGATCGCTGAACGCTATTTAACCC
GTTATTTCGTCGTCGATTTTCTTGCAGTGCTGCCTTTACCGCAGGTAAAACAGAGTGAAAAGATCCTTTGATGGTTACTAAAAATATTG
GACTTTTGGGATTGATTATGATGTTTTGTGTGGATATTACAGATTGCAGTGTGGAAGTTTCTTCACGGGTCTAAAGGATCGGATGTGTT
ACCAACGAAAACGGCATTGTTGAACATTGTAATTGTGCAGTATATTCCGAGATTCGTGAGGTTTATTCCGTTAACATCAGAGTTGAAGA
AAACTGCAGGAGCTTTTGCCGAAGGAGCTTGGGCTGGTGCTGCTTATTATCTTCTCTGGTATATGCTTGCAAGCCACGTAAGCGTTTCT
ATGTATATATGACTCTTCTTAATTAGTGTTTTGAGACATTTATTCATCATTGGTCACTTTCTGACTTTACAAACACATTTTGACAGATA
ACTGGAGCGTTTTGGTACATGTTGTCAGTGGAGCGTAACGATACTTGTTGGAGATTTGCGTGTAAAGTTCAGCCAGATCCGCGACTCTG
TGTTCAGATTCTGTATTGTGGGACTAAATTTGTGAGCAGTGGAGAAACTGAATGGATCAAAACAGTCCCTGAGCTTCTTAAGAGCAATT
GTTCTGCTAAAGCAGATGATTCAAAGTTTAACTATGGGATATACGGTCAGGCTATATCTTCAGGCATTGTATCATCAACAACATTTTTC
TCCAAGTTCTGTTATTGTCTATGGTGGGGTCTTCAAAATCTCAGGTAAGGCGGGGGCAACTCATTGTCATTGGTTCAACCGATAGCTTG
TGCAGAGTACTCTTGACATCTTTGGTGTTTTCATGAACAGCACGTTAGGTCAAGGGCTGCAGACAAGTACATTTCCAGGAGAGGTTTTG
TTTTCCATTGCGATTGCTATAGCTGGGCTTCTTCTTTTCGCGCTTCTCATCGGGAATATGCAGACTTATCTTCAGTCACTTACTGTTCG
TCTCGAGGAAATGAGGATCAAAAGACGTGACTCAGAACAGTGGATGCATCACAGGTCACTTCCACAAAATTTGAGGGAAAGAGTCAGAC
GCTATGATCAATACAAATGGTTAGAGACAAGAGGTGTAGACGAAGAAAACATAGTTCAGAGTTTACCTAAGGATCTCAGAAGAGACATC
AAACGCCATCTCTGTCTGAATTTAGTTAGGAGGGTAAGTTTTCACAATCAGAATGCTAAAATTGCTTGAGATACTAACTAACTATGTGT
TCTGTTTCTACAGGTTCCTCTGTTTGCTAATATGGATGAAAGATTACTTGACGCAATCTGTGAGAGATTAAAGCCGAGTCTTTTCACGG
AAAGCACTTACATTGTGCGTGAAGGAGACCCCGTTAACGAAATGATGTTCATAATCCGAGGCCGGTTAGAAAGTGTAACAACAGATGGG
GGAAGAAGCGGTTTCTTCAACAGAGGGTTGTTAAAAGAAGGAGACTTTTGTGGTGAAGAGCTTCTGACATGGGCTCTTGACCCTAAAGC
AGGCTCAAACTTACCTTCTTCCACAAGAACAGTGAAGGCTCTGACTGAAGTAGAGGCTTTCGCTCTTGAAGCTGAAGAGCTGAAGTTTG
TAGCTAGCCAGTGGAGGAGACTTCATAGTCGACAGGTTCAACAAACATTTAGGTTTTACTCTCAGCAATGGAGAACTTGGGCTTCTTGC
TTCATCCAAGCTGCTTGGCGTCGCTATTCAAGAAGGAAAAACGCTGAATTGAGACGGATTGAAGAAAAAGAAGAAGAATTGGGTTATGA
AGATGAATATGATGATGAGAGTGACAAGAGGCCTATGGTTATCACAAGAAGTGAGTCTTCTTCTAGACTGCGTTCAACGATATTTGCAT
CAAGATTTGCTGCTAATGCATTGAAAGGGCACAGACTTAGGAGCTCAGAGAGTTCAAAGACCTTGATAAATCTGCAAAAGCCTCCGGAA
CCTGATTTTGATGCTGAATGAGGCCTAGGCGCGGTCGCGGGGATCACTAGTGAATTCGCGGCCGCGGGGCCCGGCTGGAGCCACCCGCA
GTTCGAAAAATGAGAGCTCGAATTTCCCCGATCGTTCAAACATTTGGCAATAAAGTTTCTTAAGATTGAATCCTGTTGCCGGTCTTGCG
ATGATTATCATATAATTTCTGTTGAATTACGTTAAGCATGTAATAATTAACATGTAATGCATGACGTTATTTATGAGATGGGTTTTTAT
GATTAGAGTCCCGCAATTATACATTTAATACGCGATAGAAAACAAAATATAGCGCGCAAACTAGGATAAATTATCGCGCGCGGTGTCAT
CTATGTTACTAGATCGGGAATTCAGCTTTTGTTCCCTTTAGTGAGGGTTAATTCCGAGCTTGGCGTAATCATGGTCATAGCTGTTTCCT
GTGTGAAATTGTTATCCGCTCACAATTCCACACAACATACGAGCCGGAAGHCATAAAGTGTAAAGCCTGGGGTGCCTAATGAGTGAGCT
AACTCACATTAATTGCGTTGCGCTCACTGCCCGCTTTCCAGTCGGGAAACCTGTCGTGCCAGCTGCATTAATGAATCGGCCAACGCGCG
GGGAGAGGCGGTTTGCGTATTGGGCGCTCTTCCGCTTCCTCGCTCACTGACTCGCTGCGCTCGGTCGTTCGGCTGCGGCGAGCGGTATC
AGCTCACTCAAAGGCGGTAATACGGTTATCCACAGAATCAGGGGATAACGCAGGAAAGAACATGAAGGCCTTGACAGGATATATTGGCG
GGTAAACTAAGTCGCTGTATGTGTTTGTTTGAGATCTCATGTGAGCAAAAGGCCAGCAAAAGGCCAGGAACCGTAAAAAGGCCGCGTTG
CTGGCGTTTTTCCATAGGCTCCGCCCCCCTGACGAGCATCACAAAAATCGACGCTCAAGTCAGAGGTGGCGAAACCCGACAGGACTATA
AAGATACCAGGCGTTTCCCCCTGGAAGCTCCCTCGTGCGCTCTCCTGTTCCGACCCTGCCGCTTACCGGATACCTGTCCGCCTTTCTCC
CTTCGGGAAGCGTGGCGCTTTCTCATAGCTCACGCTGTAGGTATCTCAGTTCGGTGTAGGTCGTTCGCTCCAAGCTGGGCTGTGTGCAC
GAACCCCCCGTTCAGCCCGACCGCTGCGCCTTATCCGGTAACTATCGTCTTGAGTCCAACCCGGTAAGACACGACTTATCGCCACTGGC
AGCAGCCACTGGTAACAGGATTAGCAGAGCGAGGTATGTAGGCGGTGCTACAGAGTTCTTGAAGTGGTGGCCTAACTACGGCTACACTA
GAAGAACAGTATTTGGTATCTGCGCTCTGCTGAAGCCAGTTACCTTCGGAAGAAGAGTTGGTAGCTCTTGATCCGGCAAACAAACCACC
GCTGGTAGCGGTGGTTTTTTTGTTTGCAAGCAGCAGATTACGCGCAGAAAAAAAGGATCTCAAGAAGATCCTTTGATCTTTTCTACGGG
GTCTGACGCTCAGTGGAACGAAAACTCACGTTAAGGGATTTTGGTCATGAGATTATCAAAAAGGATCTTCACCTAGATCCTTTTAAATT
AAAAATGAAGTTTTAAATCAATCTAAAGTATATATGTGTAACATTGGTCTAGTGATTAGAAAAACTCATCGAGCATCAAATGAAACTGC
AATTTATTCATATCAGGATTATCAATACCATATTTTTGAAAAAGCCGTTTCTGTAATGAAGGAGAAAACTCACCGAGGCAGTTCCATAG
GATGGCAAGATCCTGGTATCGGTCTGCGATTCCGACTCGTCCAACATCAATACAACCTATTAATTTCCCCTCGTCAAAAATAAGGTTAT
CAAGTGAGAAATCACCATGAGTGACGACTGAATCCGGTGAGAATGGCAAAAGTTTATGCATTTCTTTCCAGACTTGTTCAACAGGCCAG
CCATTACGCTCGTCATCAAAATCACTCGCATCAACCAAACCGTTATTCATTCGTGATTGCGCCTGAGCGAGACGAAATACGCGATCGCT
GTTAAAAGGACAATTACAAACAGGAATCGAATGCAACCGGCGCAGGAACACTGCCAGCGCATCAACAATATTTTCACCTGAATCAGGAT
ATTCTTCTAATACCTGGAATGCTGTTTTCCCTGGGATCGCAGTGGTGAGTAACCATGCATCATCAGGAGTACGGATAAAATGCTTGATG
GTCGGAAGAGGCATAAATTCCGTCAGCCAGTTTAGTCTGACCATCTCATCTGTAACAACATTGGCAACGCTACCTTTGCCATGTTTCAG
AAACAACTCTGGCGCATCGGGCTTCCCATACAATCGGTAGATTGTCGCACCTGATTGCCCGACATTATCGCGAGCCCATTTATACCCAT
ATAAATCAGCATCCATGTTGGAATTTAATCGCGGCCTTGAGCAAGACGTTTCCCGTTGAATATGGCTCATAACACCCCTTGTATTACTG
TTTATGTAAGCAGACAGTTTTATTGTTCATGATGATATATTTTTATCTTGTGCAATGTAACATCAGAGATTTTGAGACACAACGTGGCT
TTGTTGAATAAATCGAACTTTTGCTGAGTTGAAGGATCAGATCACGCATCTTCCCGACAACGCAGACCGTTCCGTGGCAAAGCAAAAGT
TCAAAATCACCAACTGGTCCACCTACAACAAAGCTCTCATCAACCGTGGCTCCCTCACTTTCTGGCTGGATGATGGGGCGATTCAGGCG
ATCCCCATCCAACAGCCCGCCGTCGAGCGGGCT

Plasmid stock 1685, CNGC18promoter::intron-Flag-CNGC8(F624W)(cDNA), abbreviated as ps1685, 18p-i-Flag-CNGC8(F624W). The *CNGC18* promoter with an added intron in the 5’ UTR, extends from an SbfI site to the first SalI site, followed by a sequence encoding Flag epitope tag (green italics). A linker connects the Flag tag to the

coding sequence for *CNGC8* with an F624 to W substitution, which starts with the first ATG following an AscI site, in the context of GGCGCGCCCGGCGGAATG, and ends with a TGA upstream of a NotI site, in the context of ACAGATGACTTGAACTGA. The F to W substitution with a silent NheI diagnostic site is in the context of GCTAGCCAGTGG.

TTTTTATCCCCGGAAGCCTGTGGATAGAGGGTAGTTATCCACGTGAAACCGCTAATGCCCCGCAAAGCCTTGATTCACGGGGCTTTCCG
GCCCGCTCCAAAAACTATCCACGTGAAATCGCTAATCAGGGTACGTGAAATCGCTAATCGGAGTACGTGAAATCGCTAATAAGGTCACG
TGAAATCGCTAATCAAAAAGGCACGTGAGAACGCTAATAGCCCTTTCAGATCAACAGCTTGCAAACACCCCTCGCTCCGGCAAGTAGTT
ACAGCAAGTAGTATGTTCAATTAGCTTTTCAATTATGAATATATATATCAATTATTGGTCGCCCTTGGCTTGTGGACAATGCGCTACGC
GCACCGGCTCCGCCCGTGGACAACCGCAAGCGGTTGCCCACCGTCGAGCGCCAGCGCCTTTGCCCACAACCCGGCGGCCGGCCGCAACA
GATCGTTTTATAAATTTTTTTTTTTGAAAAAGAAAAAGCCCGAAAGGCGGCAACCTCTCGGGCTTCTGGATTTCCGATCCCCGGAATTA
GAGATCTTGGCAGGATATATTGTGGTGTAACGTTATCGATCTGGATTTTAGTACTGGATTTTGGTTTTAGGAATTAGAAATTTTATTGA
TAGAAGTATTTTACAAATACAAATACATACTAAGGGTTTCTTATATGCTCAACACATGAGCGAAACCCTATAAGAACCCTAATTTCCCT
TATCGGGAAACTACTCACACATTATTTATGGAGAAAATAGAGAGAGATAGATTTGTAGAGAGAGACTGGTGATTTCAGCGTACCGAATT
AATTCTCCCGCGACCAGCCGAGCGAGCTTAGCGAACTGTGGACGAGAACTGTGCCACCAAGCGTAAGGCCGTTCTCTCGCATTTGCCTT
GCTAGGCTCGCGCGAGTTGCTGGCTGAGGCGTTCTCGAAATCAGCTCTTGTTCGGTCGGCATCTACTCTATTCCTTTGCCCTCGGACGA
GTGCTGGGGCGTCGGTTTCCACTATCGGCGAGTACTTCTACACAGCCATCGGTCCAGACGGCCGCGCTTCTGCGGGCGATTTGTGTACG
CCCGACAGTCCCGGCTCCGGATCGGACGATTGCGTCGCATCGACCCTGCGCCCAAGCTGCATCATCGAAATTGCCGTCAACCAAGCTCT
GATAGAGTTGGTCAAGACCAATGCGGAGCATATACGCCCGGAGGCGTGGCGATCCTGCAAGCTCCGGATGCCTCCGCTCGAAGTAGCGC
GTCTGCTGCTCCATACAAGCCAACCACGGCCTCCAGAAGAAGATGTTGGCGACCTCGTATTGGGAATCCCCGAACATCGCCTCGCTCCA
GTCAATGACCGCTGTTATGCGGCCATTGTCCGTCAGGACATTGTTGGAGCCGAAATCCGCGTGCACGAGGTGCCGGACTTCGGGGCAGT
CCTCGGCCCAAAGCATCAGCTCATCGAGAGCCTGCGCGACGGACGCACTGACGGTGTCGTCCATCACAGTTTGCCAGTGATACACATGG
GGATCAGCAATCGCGCATATGAAATCACGCCATGTAGTGTATTGACCGATTCCTTGCGGTCCGAATGGGCCGAACCCGCTCGTCTGGCT
AAGATCGGCCGCAGCGATCGCATCCATAGCCTCCGCGACCGGCTGAAGAACAGCGGGCAGTTCGGTTTCAGGCAGGTCTTGCAACGTGA
CACCCTGTGCACGGCGGGAGATGCAATAGGTCAGGCTCTCGCTGAACTCCCCAATGTCAAGCACTTCCGGAATCGGGAGCGCGGCCGAT
GCAAAGTGCCGATAAACATAACGATCTTTGTAGAAACCATCGGCGCAGCTATTTACCCGCAGGACATATCCACGCCCTCCTACATCGAA
GCTGAAAGCACGAGATTCTTCGCCCTCCGAGAGCTGCATCAGGTCGGAGACGCTGTCGAACTTTTCGATCAGAAACTTCTCGACAGACG
TCGCGGTGAGTTCAGGCTTTTTCATAGGGATCAGCTTGGGCTGTCCTCTCCAAATGAAATGAACTTCCTTATATAGAGGAAGGGTCTTG
CGAAGGATAGTGGGATTGTGCGTCATCCCTTACGTCAGTGGAGATGTCACATCAATCCACTTGCTTTGAAGACGTGGTTGGAACGTCTT
CTTTTTCCACGATGCTCCTCGTGGGTGGGGGTCCATCTTTGGGACCACTGTCGGCAGAGGCATCTTGAATGATAGCCTTTCCTTTATCG
CAATGATGGCATTTGTAGGAGCCACCTTCCTTTTCTACTGTCCTTTCGATGAAGTGACAGATAGCTGGGCAATGGAATCCGAGGAGGTT
TCCCGAAATTACCCTTTGTTGAAAAGTCTCAATAGCCCTTTGGTCTTCTGAGACTGTATCTTTGACATTTTTGGAGTAGGGGTACGATA
ACATTAACGTTTACAATTTCGCGCCATTCGCCATTCAGGCTGCGCAACTGTTGGGAAGGGCGATCGGTGCGGGCCTCTTCGCTATTACG
CCAGCTGGCGAAAGGGGGATGTGCTGCAAGGCGATTAAGTTGGGTAACGCCAGGGTTTTCCCAGTCACGACGTTGTAAAACGACGGCCA
GTGAATTGTAATACGACTCACTATAGGGCGAATTGGGTACCGTTTAAACAAGCTTCCTAGCCCTGCAGGGGATCCTGGGTTTTGTATCT
AAAGATCAAAGGAATAACATTTTTGGGAGATGAGAAACTTCAAAAGATGCGATTTAGTTTTTGTGGAAAAGTGGGGAATTTAATTCGAT
TGACGTTAGGAACTTTTTCTCTATGGATTCTTTGGGTTTGTGATTAATGGCATTGAAGGTACAAAGGAAAAGGTTTGGCCAACAAAATC
AAGAACCACAAAGAGAGAGAGAGAGAGGAAACACACAGACAAAACATATGAAAGAATGATTAAGATAACTGCCACAAATATAACTGCAA
AATAACATACTCCTTAAGAAAATTAGTGGGGCCTTCTTTGTTATGGAAGAATGTGCAAATCTTTTTATATGGTTAAGTATATTAGTATT
ATTATATCAAAATTCATTTTTGACTATATATAGGTGATTGTGTCGAAATTCACGTAAGATCTTTGTAACATTTTTTCACACTAGTTAAT
TTTACATGTATTATAGATTCGCTGCAAAAAAAAAAAAAAAAAAAAAAAAAGAAAATAGAAAAATTTCTGTTTTTTCACACTAGGCCGATTT
GATTGATCAAAACCGATTTTAAAAGCTTAAACTAATAAATCGATTTCATTCACCAATTTATTAAAATTAGATCGTTTTGGAGGTTCAAG
AATATCTGGTCGCTGTTTAAGACGATATTTTGAATCTTGAACCGTTGAAACTATATATACTAGGTATATTTACTGTATGTTGTGAAAGG
GTTTACTATCACATGGCTATTATATAAGACCGTACTAACATATGCAAATATTATATGTGGATCAAAGTACCACAAATTTTGAGTGAACC
AAAAACAAAAGGTTAGAGATAGAAAAATATGCATGTAAAAAATAATAAGAGATGAAAGAAATATTAATGGGAGATGGGTGGGAAATGAA
TAGATTGGTGTCTTCGTCACTTTTACTTTTTTTTTTTTTTTATTGTTTGTTTGATAGTACCAAGAGATTCTCTATTCTATTCTTTCGTGA
TGGTTTATTTTAGGGTATATTACGTGACCTGTAGTTCATCCTCTCCAACTCTCAAACCCACAAATTATTCACTATACATATATCCAAAG
ATTATTTATTCAAAGATATGACTAAAAATCTCTATACACGTGTGTATGTAATAGAAAGGTGCAAGGATTTTGGTGTGGCACCACCTTCA
AGACTTTTTTTTTATCAAGACCTAACTCTTACCACTTTGAAAGCAGCTTATCTTATTTCTTTAGATAAAACAATTGATAAGCGAGTGCT
TTAAACTTCACAAAACTACAATCTACTTTTTATTCGATAAAACAAAAGATTTTATTAGTCTTTTTCATTAGGGTCCCAAATGATTAATA
TGTTCACGATCACGAAATTTCTTCTTGCGTCCCGTAAGCATTTATATTAATCAAACAAAAAGGAAATTTGTTGCTTCCTACAATGGGAA
GTGGGAACCTTTGCCATCTTCATAAATGTTTTAAATCTTTTTTTTTTCTTTCTAATGTGTGTTTCATAAACACCAGCGACTTTATTGAA
AAGAGGAACACGTAAATTGTATTCTCAAAGAACAATTTTTTTAGGAAGAGTAACATTTGTTTTCCTTATCTTAATATAAGCAACGAAAT
CAATCGTGCATATAAAAGTGAAATGCTATTTTGAATTTTCTAAACTTGACTAGCAAATTTTCACTAAAAAATAAAATTAAAAAGTAACG
ATAAAGTAGTTGTCGTATAGTCTACTCCACATGAATAAAATAATAAAGTAAATCAATAGAATGTAAATCTCTTAATTAATCATACTTAT
TATATAATCACAGTGGCAAAGTGGAATAAAAAAAAAAGAGGAGAATTGGATCCCGTCGGATTCTATATTTAAAGATCGCTAAACATCTC
AAATCTGCCACACGTGGTCATACTTAATAGCAGCCATTAACGAAGTAATAAATTAGTCGATAATAATGTTTAACCAACATCGTATTTCT
AGATCCATGAATTAGTTGGAGTAATTAACATCATTGTTTCTTAATTACGTTTAACAAGCATAGAGAAAACAATATTGTCACATATGTTA
GTATAAGTTATCATAATCATTTATAAAAGCTTGTTATTAATTTTTTATATTGAAATTACTACTTTGTGTCTAATCGGAAAATGATGGAG
TACTAAAATTTGAAGGACTAAAATACGTCATTATTGTTAGAGAAAACAATTACGCATAAGTGTGATTTTTTGGAAGTGGAAAAGTTAGT
ATGTCCCTCTATATGATATGATGGAAGTTGTTGTTCTTTTTGTGTGGATAAACATCATTTTTCCGTGACGTAATTTTTAATTTCAGTAT
TCATTTTATTATTCTCACTTCTTATTATCCCTTTTATATTCGCATAATTGTTTCTAAGGATTTCTATTCCCTTTTTGTTATTTTATAGG
TACACTATCTTATGGATGGTGTCAACAAGCATGTACACTATCTTCCCACTACCACATATTGTCACAGAATGTTTGGTACGCGTGCTTCT
ATATACTTTTAATAGTACATAACAACAACAAATGAATTATGTAAGACAATTTGTTAAACTGATGTCAAAAACGGAAATACTACAAAAGA
CAGTATAATTGAATTACGGGTTTAATGTGTTGACAAAATATAATAAATGTAGCTCATAAAACTTCAATAATTCACTTAAATAAATTCTA
ATGAACCTCATAACGAATTAGATATTTTAACTAATCTTATCATGGTTTTACGAATTAATTATAATTTTTTTTTTTATTCTTTGAACAAT
TGAACTAAACAATATTAAACCGGACTTTAAGTTAAAACTACTAAAAACATTATTAGTTTATCAATAGAAATGATCATAGCAATTGTATA
CATTATAATGTGTAATTTTAAAATCCTAAAGCATATATAGTATATATCCCTTTGATTTTCATAGAAAAAAAGTAGAAAAAAATAACTAT

TGGGCCGATCTATAACTATTGGGCCGGTGGGTCCAACAACTAAGAACAGTAGATATTGTGTCGATTGAAGTTTTGAGATGCAAAACGTG
TGCTGTTTTGAGAACACAAAAACAGCATAAACAAACGAGACCCGCAACTATAACATTCCGTAAATACCCTCATTCTCCCATCATATTTT
CAAAAACTACCCTTCCCCCTATTTCCATGGCCCTTCTCTCAGTTTTCCCGCGAGAAATCCTCTCCTTCTTCCATAATCATCAAAAACAA
AATGCAAAAACCCAAAAAAACAAAAAATCACACGACGCAACCACCTAGCCTCTCTGTCCATCAATCTCAATCTCTCTCTGTGTTAATGC
GGAATCCGATTTAACACCGGAATCCGCCATGGAAGGGAGGCGGTGTCGAGGTGCAGTAGTCCACCTTCACTTGCTCTCTTTTTTCCGAA
GAATCTGGTTCGTTTCTCTTTGAATTATCTCTTTCTCTGTTCTTCTTCTTTTTCTAAAGTCTCGAATATATCTTCATTATCATTACTTT
ATTTGCTTCGTTCTTCCGCAGTTTTGTGCAAAGTTTTAACGATTTTTTGGTTTTGAACGAAGGCACAGAGAGAAGGCGCGTCGAC*ATGG*
*ACTACAAAGACGATGACGACAAG*GTCGACGGCGCGCCCGGCGGAATGTCATCTAATGCTACGGGAATGAAGAAGCGATCATGTTTTGGA
TTGTTTAACGTAACTAGTCGTGGAGGAGGAAAGACAAAGAACACATCAAAATCATTTCGAGAAGGTGTTAAAATCGGATCAGAGGGTCT
AAAAACGATCGGAAAATCATTCACATCAGGTGTAACAAGAGCTGTTTTCCCTGAAGATCTAAGAGTCTCCGAGAAGAAAATCTTTGATC
CTCAAGACAAAACACTTTTATTATGGAACAGAATGTTTGTTATCTCTTGCATTCTTGCTGTCTCTGTTGATCCTCTGTTCTTCTATCTC
CCTATTGTCGATAACTCAAAGAACTGCATTGGGATTGACTCTAAATTAGCTGTGACAACTACTACTCTAAGAACGATCATCGATGTTTT
TTATCTTACACGAATGGCTCTTCAGTTCCGTACCGCTTACATTGCTCCTTCTTCTCGTGTGTTTGGAAGAGGTGAGCTTGTGATCGACC
CTGCAAAGATCGCTGAACGGTATTTGACTCGTTATTTCATTGTCGATTTCCTCGCGGTTCTTCCTTTACCTCAGATTGCTGTGTGGAAG
TTTCTTCACGGATCTAAAGGAACAGATGTATTACCAACAAAACAGGCGCTATTACACATTGTCATCACACAGTACATACCAAGATTCGT
TAGGTTTATTCCATTAACTTCAGAGCTCAAGAAAACAGCAGGAGCTTTCGCTGAAGGTGCTTGGGCTGGTGCTGCTTATTACCTCCTCT
GGTATATGCTTGCAAGTCACATAACTGGAGCGTTCTGGTACATGTTGTCAGTGGAACGTAACGATACATGCTTGAGATCCGCTTGTAAA
GTCCAGCCTGATCCCAAGGTCTGTGTTCAGATTCTTTATTGTGGCAGCAAATTAATGAGCAGTCGCGAAACCGACTGGATCAAATCAGT
CCCTGATCTTTTTAAGAACAATTGTTCTGCTAAAAGCGATGAGTCTAAATTCAACTACGGGATCTATAGCCAGGCTGTGTCTTCCGGTA
TTGTATCTTCGACAACGTTTTTCTCCAAGTTTTGTTATTGTCTATGGTGGGGTCTCCAAAATCTCAGCACGTTAGGTCAAGGGCTGCAA
ACAAGTACATATCCAGGAGAAGTTTTGTTTTCAATTGCAATTGCTGTAGCTGGACTTCTTTTGTTTGCTCTTCTCATTGGGAATATGCA
AACTTATCTTCAGTCACTTACGGTTCGCTTAGAGGAAATGAGGATTAAAAGACGTGATTCCGAACAGTGGATGCATCACAGGTCACTTC
CACAAAACCTGAGGGAACGAGTCAGACGTTATGATCAATACAAATGGTTGGAAACAAGAGGAGTCGACGAAGAAAACATAGTCCAGAGT
TTACCAAAAGATCTTAGAAGAGACATCAAACGCCATCTCTGTCTAAATTTAGTTCGCAGGGTTCCTCTGTTTGCTAATATGGATGAGAG
ATTACTAGACGCAATATGTGAGAGACTAAAGCCAAGTCTATACACAGAGAGCACTTACATAGTGCGAGAAGGAGACCCGGTTAACGAAA
TGCTCTTCATAATCCGAGGCCGGTTAGAGAGTGTAACAACAGATGGTGGAAGAAGCGGTTTCTTTAACAGAGGTTTATTGAAAGAAGGT
GACTTTTGTGGTGAAGAGCTTCTGACTTGGGCACTTGACCCTAAAGCAGGCTCAAACTTACCTTCTTCCACACGAACAGTGAAGGCTCT
AACTGAAGTAGAGGCTTTCGCTTTAGAAGCGGAAGAGCTCAAGTTTGTGGCTAGCCAGTGGAGGCGTCTTCACAGTCGTCAGGTTCAAC
AAACTTTCAGGTTTTACTCTCAACAATGGAGGACTTGGGCTGCTTGTTTCATCCAAGCCGCTTGGCGTAGACATTTAAGAAGGAAAATC
GCAGAGCTTAGACGTAAAGAAGAAGAAGAAGAAGAAATGGATTATGAAGATGATGAATATTATGATGATAATATGGGTGGTATGGTTAC
AAGGAGTGATAGTTCTGTTGGATCAAGTTCTACATTACGTTCCACGGTATTTGCATCAAGATTTGCTGCTAACGCGCTTAAGGGTCATA
AACTAAGGGTCACCGAGAGTTCAAAGAGTTTAATGAATTTAACAAAGCCATCAGAACCTGACTTTGAGGCTCTTGATACAGATGACTTG
AACTGAGGCCTAGGCGCGGCCGCGAATTCACTAGTGATTCCCCGCGGCCGCGGGGCCCGGCTGGAGCCACCCGCAGTTCGAAAAATGAG
AGCTCGAATTTCCCCGATCGTTCAAACATTTGGCAATAAAGTTTCTTAAGATTGAATCCTGTTGCCGGTCTTGCGATGATTATCATATA
ATTTCTGTTGAATTACGTTAAGCATGTAATAATTAACATGTAATGCATGACGTTATTTATGAGATGGGTTTTTATGATTAGAGTCCCGC
AATTATACATTTAATACGCGATAGAAAACAAAATATAGCGCGCAAACTAGGATAAATTATCGCGCGCGGTGTCATCTATGTTACTAGAT
CGGGAATTCAGCTTTTGTTCCCTTTAGTGAGGGTTAATTCCGAGCTTGGCGTAATCATGGTCATAGCTGTTTCCTGTGTGAAATTGTTA
TCCGCTCACAATTCCACACAACATACGAGCCGGAAGCATAAAGTGTAAAGCCTGGGGTGCCTAATGAGTGAGCTAACTCACATTAATTG
CGTTGCGCTCACTGCCCGCTTTCCAGTCGGGAAACCTGTCGTGCCAGCTGCATTAATGAATCGGCCAACGCGCGGGGAGAGGCGGTTTG
CGTATTGGGCGCTCTTCCGCTTCCTCGCTCACTGACTCGCTGCGCTCGGTCGTTCGGCTGCGGCGAGCGGTATCAGCTCACTCAAAGGC
GGTAATACGGTTATCCACAGAATCAGGGGATAACGCAGGAAAGAACATGAAGGCCTTGACAGGATATATTGGCGGGTAAACTAAGTCGC
TGTATGTGTTTGTTTGAGATCTCATGTGAGCAAAAGGCCAGCAAAAGGCCAGGAACCGTAAAAAGGCCGCGTTGCTGGCGTTTTTCCAT
AGGCTCCGCCCCCCTGACGAGCATCACAAAAATCGACGCTCAAGTCAGAGGTGGCGAAACCCGACAGGACTATAAAGATACCAGGCGTT
TCCCCCTGGAAGCTCCCTCGTGCGCTCTCCTGTTCCGACCCTGCCGCTTACCGGATACCTGTCCGCCTTTCTCCCTTCGGGAAGCGTGG
CGCTTTCTCATAGCTCACGCTGTAGGTATCTCAGTTCGGTGTAGGTCGTTCGCTCCAAGCTGGGCTGTGTGCACGAACCCCCCGTTCAG
CCCGACCGCTGCGCCTTATCCGGTAACTATCGTCTTGAGTCCAACCCGGTAAGACACGACTTATCGCCACTGGCAGCAGCCACTGGTAA
CAGGATTAGCAGAGCGAGGTATGTAGGCGGTGCTACAGAGTTCTTGAAGTGGTGGCCTAACTACGGCTACACTAGAAGAACAGTATTTG
GTATCTGCGCTCTGCTGAAGCCAGTTACCTTCGGAAGAAGAGTTGGTAGCTCTTGATCCGGCAAACAAACCACCGCTGGTAGCGGTGGT
TTTTTTGTTTGCAAGCAGCAGATTACGCGCAGAAAAAAAGGATCTCAAGAAGATCCTTTGATCTTTTCTACGGGGTCTGACGCTCAGTG
GAACGAAAACTCACGTTAAGGGATTTTGGTCATGAGATTATCAAAAAGGATCTTCACCTAGATCCTTTTAAATTAAAAATGAAGTTTTA
AATCAATCTAAAGTATATATGTGTAACATTGGTCTAGTGATTAGAAAAACTCATCGAGCATCAAATGAAACTGCAATTTATTCATATCA
GGATTATCAATACCATATTTTTGAAAAAGCCGTTTCTGTAATGAAGGAGAAAACTCACCGAGGCAGTTCCATAGGATGGCAAGATCCTG
GTATCGGTCTGCGATTCCGACTCGTCCAACATCAATACAACCTATTAATTTCCCCTCGTCAAAAATAAGGTTATCAAGTGAGAAATCAC
CATGAGTGACGACTGAATCCGGTGAGAATGGCAAAAGTTTATGCATTTCTTTCCAGACTTGTTCAACAGGCCAGCCATTACGCTCGTCA
TCAAAATCACTCGCATCAACCAAACCGTTATTCATTCGTGATTGCGCCTGAGCGAGACGAAATACGCGATCGCTGTTAAAAGGACAATT
ACAAACAGGAATCGAATGCAACCGGCGCAGGAACACTGCCAGCGCATCAACAATATTTTCACCTGAATCAGGATATTCTTCTAATACCT
GGAATGCTGTTTTCCCTGGGATCGCAGTGGTGAGTAACCATGCATCATCAGGAGTACGGATAAAATGCTTGATGGTCGGAAGAGGCATA
AATTCCGTCAGCCAGTTTAGTCTGACCATCTCATCTGTAACAACATTGGCAACGCTACCTTTGCCATGTTTCAGAAACAACTCTGGCGC
ATCGGGCTTCCCATACAATCGGTAGATTGTCGCACCTGATTGCCCGACATTATCGCGAGCCCATTTATACCCATATAAATCAGCATCCA
TGTTGGAATTTAATCGCGGCCTTGAGCAAGACGTTTCCCGTTGAATATGGCTCATAACACCCCTTGTATTACTGTTTATGTAAGCAGAC
AGTTTTATTGTTCATGATGATATATTTTTATCTTGTGCAATGTAACATCAGAGATTTTGAGACACAACGTGGCTTTGTTGAATAAATCG
AACTTTTGCTGAGTTGAAGGATCAGATCACGCATCTTCCCGACAACGCAGACCGTTCCGTGGCAAAGCAAAAGTTCAAAATCACCAACT
GGTCCACCTACAACAAAGCTCTCATCAACCGTGGCTCCCTCACTTTCTGGCTGGATGATGGGGCGATTCAGGCGATCCCCATCCAACAG
CCCGCCGTCGAGCGGGCT

Plasmid stock 1687, CNGC18promoter::intron-Flag-CNGC8(cDNA), abbreviated as ps1687, 18p-i-Flag-CNGC8. The *CNGC18* promoter with an added intron in the 5’ UTR, extends from an SbfI site to the first SalI site, followed by a sequence encoding Flag epitope tag (green italics). A linker connects the Flag tag to the coding sequence for *CNGC8*, which starts with the first ATG following an AscI site, in the

context of GGCGCGCCCGGCGGAATG, and ends with a TGA upstream of a NotI site, in the context of ACAGATGACTTGAACTGA.

TTTTTATCCCCGGAAGCCTGTGGATAGAGGGTAGTTATCCACGTGAAACCGCTAATGCCCCGCAAAGCCTTGATTCACGGGGCTTTCCG
GCCCGCTCCAAAAACTATCCACGTGAAATCGCTAATCAGGGTACGTGAAATCGCTAATCGGAGTACGTGAAATCGCTAATAAGGTCACG
TGAAATCGCTAATCAAAAAGGCACGTGAGAACGCTAATAGCCCTTTCAGATCAACAGCTTGCAAACACCCCTCGCTCCGGCAAGTAGTT
ACAGCAAGTAGTATGTTCAATTAGCTTTTCAATTATGAATATATATATCAATTATTGGTCGCCCTTGGCTTGTGGACAATGCGCTACGC
GCACCGGCTCCGCCCGTGGACAACCGCAAGCGGTTGCCCACCGTCGAGCGCCAGCGCCTTTGCCCACAACCCGGCGGCCGGCCGCAACA
GATCGTTTTATAAATTTTTTTTTTTGAAAAAGAAAAAGCCCGAAAGGCGGCAACCTCTCGGGCTTCTGGATTTCCGATCCCCGGAATTA
GAGATCTTGGCAGGATATATTGTGGTGTAACGTTATCGATCTGGATTTTAGTACTGGATTTTGGTTTTAGGAATTAGAAATTTTATTGA
TAGAAGTATTTTACAAATACAAATACATACTAAGGGTTTCTTATATGCTCAACACATGAGCGAAACCCTATAAGAACCCTAATTTCCCT
TATCGGGAAACTACTCACACATTATTTATGGAGAAAATAGAGAGAGATAGATTTGTAGAGAGAGACTGGTGATTTCAGCGTACCGAATT
AATTCTCCCGCGACCAGCCGAGCGAGCTTAGCGAACTGTGGACGAGAACTGTGCCACCAAGCGTAAGGCCGTTCTCTCGCATTTGCCTT
GCTAGGCTCGCGCGAGTTGCTGGCTGAGGCGTTCTCGAAATCAGCTCTTGTTCGGTCGGCATCTACTCTATTCCTTTGCCCTCGGACGA
GTGCTGGGGCGTCGGTTTCCACTATCGGCGAGTACTTCTACACAGCCATCGGTCCAGACGGCCGCGCTTCTGCGGGCGATTTGTGTACG
CCCGACAGTCCCGGCTCCGGATCGGACGATTGCGTCGCATCGACCCTGCGCCCAAGCTGCATCATCGAAATTGCCGTCAACCAAGCTCT
GATAGAGTTGGTCAAGACCAATGCGGAGCATATACGCCCGGAGGCGTGGCGATCCTGCAAGCTCCGGATGCCTCCGCTCGAAGTAGCGC
GTCTGCTGCTCCATACAAGCCAACCACGGCCTCCAGAAGAAGATGTTGGCGACCTCGTATTGGGAATCCCCGAACATCGCCTCGCTCCA
GTCAATGACCGCTGTTATGCGGCCATTGTCCGTCAGGACATTGTTGGAGCCGAAATCCGCGTGCACGAGGTGCCGGACTTCGGGGCAGT
CCTCGGCCCAAAGCATCAGCTCATCGAGAGCCTGCGCGACGGACGCACTGACGGTGTCGTCCATCACAGTTTGCCAGTGATACACATGG
GGATCAGCAATCGCGCATATGAAATCACGCCATGTAGTGTATTGACCGATTCCTTGCGGTCCGAATGGGCCGAACCCGCTCGTCTGGCT
AAGATCGGCCGCAGCGATCGCATCCATAGCCTCCGCGACCGGCTGAAGAACAGCGGGCAGTTCGGTTTCAGGCAGGTCTTGCAACGTGA
CACCCTGTGCACGGCGGGAGATGCAATAGGTCAGGCTCTCGCTGAACTCCCCAATGTCAAGCACTTCCGGAATCGGGAGCGCGGCCGAT
GCAAAGTGCCGATAAACATAACGATCTTTGTAGAAACCATCGGCGCAGCTATTTACCCGCAGGACATATCCACGCCCTCCTACATCGAA
GCTGAAAGCACGAGATTCTTCGCCCTCCGAGAGCTGCATCAGGTCGGAGACGCTGTCGAACTTTTCGATCAGAAACTTCTCGACAGACG
TCGCGGTGAGTTCAGGCTTTTTCATAGGGATCAGCTTGGGCTGTCCTCTCCAAATGAAATGAACTTCCTTATATAGAGGAAGGGTCTTG
CGAAGGATAGTGGGATTGTGCGTCATCCCTTACGTCAGTGGAGATGTCACATCAATCCACTTGCTTTGAAGACGTGGTTGGAACGTCTT
CTTTTTCCACGATGCTCCTCGTGGGTGGGGGTCCATCTTTGGGACCACTGTCGGCAGAGGCATCTTGAATGATAGCCTTTCCTTTATCG
CAATGATGGCATTTGTAGGAGCCACCTTCCTTTTCTACTGTCCTTTCGATGAAGTGACAGATAGCTGGGCAATGGAATCCGAGGAGGTT
TCCCGAAATTACCCTTTGTTGAAAAGTCTCAATAGCCCTTTGGTCTTCTGAGACTGTATCTTTGACATTTTTGGAGTAGGGGTACGATA
ACATTAACGTTTACAATTTCGCGCCATTCGCCATTCAGGCTGCGCAACTGTTGGGAAGGGCGATCGGTGCGGGCCTCTTCGCTATTACG
CCAGCTGGCGAAAGGGGGATGTGCTGCAAGGCGATTAAGTTGGGTAACGCCAGGGTTTTCCCAGTCACGACGTTGTAAAACGACGGCCA
GTGAATTGTAATACGACTCACTATAGGGCGAATTGGGTACCGTTTAAACAAGCTTCCTAGCCCTGCAGGGGATCCTGGGTTTTGTATCT
AAAGATCAAAGGAATAACATTTTTGGGAGATGAGAAACTTCAAAAGATGCGATTTAGTTTTTGTGGAAAAGTGGGGAATTTAATTCGAT
TGACGTTAGGAACTTTTTCTCTATGGATTCTTTGGGTTTGTGATTAATGGCATTGAAGGTACAAAGGAAAAGGTTTGGCCAACAAAATC
AAGAACCACAAAGAGAGAGAGAGAGAGGAAACACACAGACAAAACATATGAAAGAATGATTAAGATAACTGCCACAAATATAACTGCAA
AATAACATACTCCTTAAGAAAATTAGTGGGGCCTTCTTTGTTATGGAAGAATGTGCAAATCTTTTTATATGGTTAAGTATATTAGTATT
ATTATATCAAAATTCATTTTTGACTATATATAGGTGATTGTGTCGAAATTCACGTAAGATCTTTGTAACATTTTTTCACACTAGTTAAT
TTTACATGTATTATAGATTCGCTGCAAAAAAAAAAAAAAAAAAAAAAAAGAAAATAGAAAAATTTCTGTTTTTTCACACTAGGCCGATTT
GATTGATCAAAACCGATTTTAAAAGCTTAAACTAATAAATCGATTTCATTCACCAATTTATTAAAATTAGATCGTTTTGGAGGTTCAAG
AATATCTGGTCGCTGTTTAAGACGATATTTTGAATCTTGAACCGTTGAAACTATATATACTAGGTATATTTACTGTATGTTGTGAAAGG
GTTTACTATCACATGGCTATTATATAAGACCGTACTAACATATGCAAATATTATATGTGGATCAAAGTACCACAAATTTTGAGTGAACC
AAAAACAAAAGGTTAGAGATAGAAAAATATGCATGTAAAAAATAATAAGAGATGAAAGAAATATTAATGGGAGATGGGTGGAAATGAA
TAGATTGGTGTCTTCGTCACTTTTACTTTTTTTTTTTTTTATTGTTTGTTTGATAGTACCAAGAGATTCTCTATTCTATTCTTTCGTGA
TGGTTTATTTTAGGGTATATTACGTGACCTGTAGTTCATCCTCTCCAACTCTCAAACCCACAAATTATTCACTATACATATATCCAAAG
ATTATTTATTCAAAGATATGACTAAAAATCTCTATACACGTGTGTATGTAATAGAAAGGTGCAAGGATTTTGGTGTGGCACCACCTTCA
AGACTTTTTTTTTATCAAGACCTAACTCTTACCACTTTGAAAGCAGCTTATCTTATTTCTTTAGATAAAACAATTGATAAGCGAGTGCT
TTAAACTTCACAAAACTACAATCTACTTTTTATTCGATAAAACAAAAGATTTTATTAGTCTTTTTCATTAGGGTCCCAAATGATTAATA
TGTTCACGATCACGAAATTTCTTCTTGCGTCCCGTAAGCATTTATATTAATCAAACAAAAAGGAAATTTGTTGCTTCCTACAATGGGAA
GTGGGAACCTTTGCCATCTTCATAAATGTTTTAAATCTTTTTTTTTTTCTTTCTAATGTGTGTTTCATAAACACCAGCGACTTTATTGAA
AAGAGGAACACGTAAATTGTATTCTCAAAGAACAATTTTTTTAGGAAGAGTAACATTTGTTTTCCTTATCTTAATATAAGCAACGAAAT
CAATCGTGCATATAAAAGTGAAATGCTATTTTGAATTTTCTAAACTTGACTAGCAAATTTTCACTAAAAAATAAAATTAAAAAGTAACG
ATAAAGTAGTTGTCGTATAGTCTACTCCACATGAATAAAATAATAAAGTAAATCAATAGAATGTAAATCTCTTAATTAATCATACTTAT
TATATAATCACAGTGGCAAAGTGGAATAAAAAAAAAAGAGGAGAATTGGATCCCGTCGGATTCTATATTTAAAGATCGCTAAACATCTC
AAATCTGCCACACGTGGTCATACTTAATAGCAGCCATTAACGAAGTAATAAATTAGTCGATAATAATGTTTAACCAACATCGTATTTCT
AGATCCATGAATTAGTTGGAGTAATTAACATCATTGTTTCTTAATTACGTTTAACAAGCATAGAGAAAACAATATTGTCACATATGTTA
GTATAAGTTATCATAATCATTTATAAAAGCTTGTTATTAATTTTTTATATTGAAATTACTACTTTGTGTCTAATCGGAAAATGATGGAG
TACTAAAATTTGAAGGACTAAAATACGTCATTATTGTTAGAGAAAACAATTACGCATAAGTGTGATTTTTTGGAAGTGGAAAAGTTAGT
ATGTCCCTCTATATGATATGATGGAAGTTGTTGTTCTTTTTGTGTGGATAAACATCATTTTTCCGTGACGTAATTTTTAATTTCAGTAT
TCATTTTATTATTCTCACTTCTTATTATCCCTTTTATATTCGCATAATTGTTTCTAAGGATTTCTATTCCCTTTTTGTTATTTTATAGG
TACACTATCTTATGGATGGTGTCAACAAGCATGTACACTATCTTCCCACTACCACATATTGTCACAGAATGTTTGGTACGCGTGCTTCT
ATATACTTTTAATAGTACATAACAACAACAAATGAATTATGTAAGACAATTTGTTAAACTGATGTCAAAAACGGAAATACTACAAAAGA
CAGTATAATTGAATTACGGGTTTAATGTGTTGACAAAATATAATAAATGTAGCTCATAAAACTTCAATAATTCACTTAAATAAATTCTA
ATGAACCTCATAACGAATTAGATATTTTAACTAATCTTATCATGGTTTTACGAATTAATTATAATTTTTTTTTTTATTCTTTGAACAAT
TGAACTAAACAATATTAAACCGGACTTTAAGTTAAAACTACTAAAAACATTATTAGTTTATCAATAGAAATGATCATAGCAATTGTATA
CATTATAATGTGTAATTTTAAAATCCTAAAGCATATATAGTATATATCCCTTTGATTTTCATAGAAAAAAAGTAGAAAAAAATAACTAT
TGGGCCGATCTATAACTATTGGGCCGGTGGGTCCAACAACTAAGAACAGTAGATATTGTGTCGATTGAAGTTTTGAGATGCAAAACGTG
TGCTGTTTTGAGAACACAAAAACAGCATAAACAAACGAGACCCGCAACTATAACATTCCGTAAATACCCTCATTCTCCCATCATATTTT
CAAAAACTACCCTTCCCCCTATTTCCATGGCCCTTCTCTCAGTTTTCCCGCGAGAAATCCTCTCCTTCTTCCATAATCATCAAAAACAA
AATGCAAAAACCCAAAAAAACAAAAAATCACACGACGCAACCACCTAGCCTCTCTGTCCATCAATCTCAATCTCTCTCTGTGTTAATGC

GGAATCCGATTTAACACCGGAATCCGCCATGGAAGGGAGGCGGTGTCGAGGTGCAGTAGTCCACCTTCACTTGCTCTCTTTTTTCCGAA
GAATCTGGTTCGTTTCTCTTTGAATTATCTCTTTCTCTGTTCTTCTTCTTTTTCTAAAGTCTCGAATATATCTTCATTATCATTACTTT
ATTTGCTTCGTTCTTCCGCAGTTTTGTGCAAAGTTTTAACGATTTTTTGGTTTTGAACGAAGGCACAGAGAGAAGGCGCGTCGAC*ATGG*
*ACTACAAAGACGATGACGACAAG*GTCGACGGCGCGCCCGGCGGAATGTCATCTAATGCTACGGGAATGAAGAAGCGATCATGTTTTGGA
TTGTTTAACGTAACTAGTCGTGGAGGAGGAAAGACAAAGAACACATCAAAATCATTTCGAGAAGGTGTTAAAATCGGATCAGAGGGTCT
AAAAACGATCGGAAAATCATTCACATCAGGTGTAACAAGAGCTGTTTTCCCTGAAGATCTAAGAGTCTCCGAGAAGAAAATCTTTGATC
CTCAAGACAAAACACTTTTATTATGGAACAGAATGTTTGTTATCTCTTGCATTCTTGCTGTCTCTGTTGATCCTCTGTTCTTCTATCTC
CCTATTGTCGATAACTCAAAGAACTGCATTGGGATTGACTCTAAATTAGCTGTGACAACTACTACTCTAAGAACGATCATCGATGTTTT
TTATCTTACACGAATGGCTCTTCAGTTCCGTACCGCTTACATTGCTCCTTCTTCTCGTGTGTTTGGAAGAGGTGAGCTTGTGATCGACC
CTGCAAAGATCGCTGAACGGTATTTGACTCGTTATTTCATTGTCGATTTCCTCGCGGTTCTTCCTTTACCTCAGATTGCTGTGTGGAAG
TTTCTTCACGGATCTAAAGGAACAGATGTATTACCAACAAAACAGGCGCTATTACACATTGTCATCACACAGTACATACCAAGATTCGT
TAGGTTTATTCCATTAACTTCAGAGCTCAAGAAAACAGCAGGAGCTTTCGCTGAAGGTGCTTGGGCTGGTGCTGCTTATTACCTCCTCT
GGTATATGCTTGCAAGTCACATAACTGGAGCGTTCTGGTACATGTTGTCAGTGGAACGTAACGATACATGCTTGAGATCCGCTTGTAAA
GTCCAGCCTGATCCCAAGGTCTGTGTTCAGATTCTTTATTGTGGCAGCAAATTAATGAGCAGTCGCGAAACCGACTGGATCAAATCAGT
CCCTGATCTTTTTAAGAACAATTGTTCTGCTAAAAGCGATGAGTCTAAATTCAACTACGGGATCTATAGCCAGGCTGTGTCTTCCGGTA
TTGTATCTTCGACAACGTTTTTCTCCAAGTTTTGTTATTGTCTATGGTGGGGTCTCCAAAATCTCAGCACGTTAGGTCAAGGGCTGCAA
ACAAGTACATATCCAGGAGAAGTTTTGTTTTCAATTGCAATTGCTGTAGCTGGACTTCTTTTGTTTGCTCTTCTCATTGGGAATATGCA
AACTTATCTTCAGTCACTTACGGTTCGCTTAGAGGAAATGAGGATTAAAAGACGTGATTCCGAACAGTGGATGCATCACAGGTCACTTC
CACAAAACCTGAGGGAACGAGTCAGACGTTATGATCAATACAAATGGTTGGAAACAAGAGGAGTCGACGAAGAAAACATAGTCCAGAGT
TTACCAAAAGATCTTAGAAGAGACATCAAACGCCATCTCTGTCTAAATTTAGTTCGCAGGGTTCCTCTGTTTGCTAATATGGATGAGAG
ATTACTAGACGCAATATGTGAGAGACTAAAGCCAAGTCTATACACAGAGAGCACTTACATAGTGCGAGAAGGAGACCCGGTTAACGAAA
TGCTCTTCATAATCCGAGGCCGGTTAGAGAGTGTAACAACAGATGGTGGAAGAAGCGGTTTCTTTAACAGAGGTTTATTGAAAGAAGGT
GACTTTTGTGGTGAAGAGCTTCTGACTTGGGCACTTGACCCTAAAGCAGGCTCAAACTTACCTTCTTCCACACGAACAGTGAAGGCTCT
AACTGAAGTAGAGGCTTTCGCTTTAGAAGCGGAAGAGCTCAAGTTTGTGGCTAGTCAGTTCAGGCGTCTTCACAGTCGTCAGGTTCAAC
AAACTTTCAGGTTTTACTCTCAACAATGGAGGACTTGGGCTGCTTGTTTCATCCAAGCCGCTTGGCGTAGACATTTAAGAAGGAAAATC
GCAGAGCTTAGACGTAAAGAAGAAGAAGAAGAAGAAATGGATTATGAAGATGATGAATATTATGATGATAATATGGGTGGTATGGTTAC
AAGGAGTGATAGTTCTGTTGGATCAAGTTCTACATTACGTTCCACGGTATTTGCATCAAGATTTGCTGCTAACGCGCTTAAGGGTCATA
AACTAAGGGTCACCGAGAGTTCAAAGAGTTTAATGAATTTAACAAAGCCATCAGAACCTGACTTTGAGGCTCTTGATACAGATGACTTG
AACTGAGGCCTAGGCGCGGCCGCGAATTCACTAGTGATTCCCCGCGGCCGCGGGGCCCGGCTGGAGCCACCCGCAGTTCGAAAAATGAG
AGCTCGAATTTCCCCGATCGTTCAAACATTTGGCAATAAAGTTTCTTAAGATTGAATCCTGTTGCCGGTCTTGCGATGATTATCATATA
ATTTCTGTTGAATTACGTTAAGCATGTAATAATTAACATGTAATGCATGACGTTATTTATGAGATGGGTTTTTATGATTAGAGTCCCGC
AATTATACATTTAATACGCGATAGAAAACAAAATATAGCGCGCAAACTAGGATAAATTATCGCGCGCGGTGTCATCTATGTTACTAGAT
CGGGAATTCAGCTTTTGTTCCCTTTAGTGAGGGTTAATTCCGAGCTTGGCGTAATCATGGTCATAGCTGTTTCCTGTGTGAAATTGTTA
TCCGCTCACAATTCCACACAACATACGAGCCGGAAGCATAAAGTGTAAAGCCTGGGGTGCCTAATGAGTGAGCTAACTCACATTAATTG
CGTTGCGCTCACTGCCCGCTTTCCAGTCGGGAAACCTGTCGTGCCAGCTGCATTAATGAATCGGCCAACGCGCGGGGAGAGGCGGTTTG
CGTATTGGGCGCTCTTCCGCTTCCTCGCTCACTGACTCGCTGCGCTCGGTCGTTCGGCTGCGGCGAGCGGTATCAGCTCACTCAAAGGC
GGTAATACGGTTATCCACAGAATCAGGGGATAACGCAGGAAAGAACATGAAGGCCTTGACAGGATATATTGGCGGGTAAACTAAGTCGC
TGTATGTGTTTGTTTGAGATCTCATGTGAGCAAAAGGCCAGCAAAAGGCCAGGAACCGTAAAAAGGCCGCGTTGCTGGCGTTTTTCCAT
AGGCTCCGCCCCCCTGACGAGCATCACAAAAATCGACGCTCAAGTCAGAGGTGGCGAAACCCGACAGGACTATAAAGATACCAGGCGTT
TCCCCCTGGAAGCTCCCTCGTGCGCTCTCCTGTTCCGACCCTGCCGCTTACCGGATACCTGTCCGCCTTTCTCCCTTCGGGAAGCGTGG
CGCTTTCTCATAGCTCACGCTGTAGGTATCTCAGTTCGGTGTAGGTCGTTCGCTCCAAGCTGGGCTGTGTGCACGAACCCCCCGTTCAG
CCCGACCGCTGCGCCTTATCCGGTAACTATCGTCTTGAGTCCAACCCGGTAAGACACGACTTATCGCCACTGGCAGCAGCCACTGGTAA
CAGGATTAGCAGAGCGAGGTATGTAGGCGGTGCTACAGAGTTCTTGAAGTGGTGGCCTAACTACGGCTACACTAGAAGAACAGTATTTG
GTATCTGCGCTCTGCTGAAGCCAGTTACCTTCGGAAGAAGAGTTGGTAGCTCTTGATCCGGCAAACAAACCACCGCTGGTAGCGGTGGT
TTTTTTGTTTGCAAGCAGCAGATTACGCGCAGAAAAAAAGGATCTCAAGAAGATCCTTTGATCTTTTCTACGGGGTCTGACGCTCAGTG
GAACGAAAACTCACGTTAAGGGATTTTGGTCATGAGATTATCAAAAAGGATCTTCACCTAGATCCTTTTAAATTAAAAATGAAGTTTTA
AATCAATCTAAAGTATATATGTGTAACATTGGTCTAGTGATTAGAAAAACTCATCGAGCATCAAATGAAACTGCAATTTATTCATATCA
GGATTATCAATACCATATTTTTGAAAAAGCCGTTTCTGTAATGAAGGAGAAAACTCACCGAGGCAGTTCCATAGGATGGCAAGATCCTG
GTATCGGTCTGCGATTCCGACTCGTCCAACATCAATACAACCTATTAATTTCCCCTCGTCAAAAATAAGGTTATCAAGTGAGAAATCAC
CATGAGTGACGACTGAATCCGGTGAGAATGGCAAAAGTTTATGCATTTCTTTCCAGACTTGTTCAACAGGCCAGCCATTACGCTCGTCA
TCAAAATCACTCGCATCAACCAAACCGTTATTCATTCGTGATTGCGCCTGAGCGAGACGAAATACGCGATCGCTGTTAAAAGGACAATT
ACAAACAGGAATCGAATGCAACCGGCGCAGGAACACTGCCAGCGCATCAACAATATTTTCACCTGAATCAGGATATTCTTCTAATACCT
GGAATGCTGTTTTCCCTGGGATCGCAGTGGTGAGTAACCATGCATCATCAGGAGTACGGATAAAATGCTTGATGGTCGGAAGAGGCATA
AATTCCGTCAGCCAGTTTAGTCTGACCATCTCATCTGTAACAACATTGGCAACGCTACCTTTGCCATGTTTCAGAAACAACTCTGGCGC
ATCGGGCTTCCCATACAATCGGTAGATTGTCGCACCTGATTGCCCGACATTATCGCGAGCCCATTTATACCCATATAAATCAGCATCCA
TGTTGGAATTTAATCGCGGCCTTGAGCAAGACGTTTCCCGTTGAATATGGCTCATAACACCCCTTGTATTACTGTTTATGTAAGCAGAC
AGTTTTATTGTTCATGATGATATATTTTTATCTTGTGCAATGTAACATCAGAGATTTTGAGACACAACGTGGCTTTGTTGAATAAATCG
AACTTTTGCTGAGTTGAAGGATCAGATCACGCATCTTCCCGACAACGCAGACCGTTCCGTGGCAAAGCAAAAGTTCAAAATCACCAACT
GGTCCACCTACAACAAAGCTCTCATCAACCGTGGCTCCCTCACTTTCTGGCTGGATGATGGGGCGATTCAGGCGATCCCCATCCAACAG
CCCGCCGTCGAGCGGGCT

Plasmid stock 1692, CNGC18promoter::intron-Flag-CNGC7(genomic DNA), abbreviated as ps1692, 18p-i-Flag-CNGC7. The *CNGC18* promoter with an added intron in the 5' UTR, extends from an SbfI site to the first SalI site, followed by a sequence encoding Flag epitope tag (green italics). A linker connects the Flag tag to the coding sequence for *CNGC7*, which starts with the first ATG following an AscI site, in the context of GGCGCGCCCGGCGGAATG, and ends with a TGA upstream of a NotI site, in the context of GATTTTGATGCTGAATGA.

TTTTTATCCCCGGAAGCCTGTGGATAGAGGGTAGTTATCCACGTGAAACCGCTAATGCCCCGCAAAGCCTTGATTCACGGGGCTTTCCG
GCCCGCTCCAAAAACTATCCACGTGAAATCGCTAATCAGGGTACGTGAAATCGCTAATCGGAGTACGTGAAATCGCTAATAAGGTCACG
TGAAATCGCTAATCAAAAAGGCACGTGAGAACGCTAATAGCCCTTTCAGATCAACAGCTTGCAAACACCCCTCGCTCCGGCAAGTAGTT
ACAGCAAGTAGTATGTTCAATTAGCTTTTCAATTATGAATATATATATCAATTATTGGTCGCCCTTGGCTTGTGGACAATGCGCTACGC
GCACCGGCTCCGCCCGTGGACAACCGCAAGCGGTTGCCCACCGTCGAGCGCCAGCGCCTTTGCCCACAACCCGGCGGCCGGCCGCAACA
GATCGTTTTATAAATTTTTTTTTTTGAAAAAGAAAAAGCCCGAAAGGCGGCAACCTCTCGGGCTTCTGGATTTCCGATCCCCGGAATTA
GAGATCTTGGCAGGATATATTGTGGTGTAACGTTATCGATCTGGATTTTAGTACTGGATTTTGGTTTTAGGAATTAGAAATTTTATTGA
TAGAAGTATTTTACAAATACAAATACATACTAAGGGTTTCTTATATGCTCAACACATGAGCGAAACCCTATAAGAACCCTAATTTCCCT
TATCGGGAAACTACTCACACATTATTTATGGAGAAAATAGAGAGAGATAGATTTGTAGAGAGAGACTGGTGATTTCAGCGTACCGAATT
AATTCTCCCGCGACCAGCCGAGCGAGCTTAGCGAACTGTGGACGAGAACTGTGCCACCAAGCGTAAGGCCGTTCTCTCGCATTTGCCTT
GCTAGGCTCGCGCGAGTTGCTGGCTGAGGCGTTCTCGAAATCAGCTCTTGTTCGGTCGGCATCTACTCTATTCCTTTGCCCTCGGACGA
GTGCTGGGGCGTCGGTTTCCACTATCGGCGAGTACTTCTACACAGCCATCGGTCCAGACGGCCGCGCTTCTGCGGGCGATTTGTGTACG
CCCGACAGTCCCGGCTCCGGATCGGACGATTGCGTCGCATCGACCCTGCGCCCAAGCTGCATCATCGAAATTGCCGTCAACCAAGCTCT
GATAGAGTTGGTCAAGACCAATGCGGAGCATATACGCCCGGAGGCGTGGCGATCCTGCAAGCTCCGGATGCCTCCGCTCGAAGTAGCGC
GTCTGCTGCTCCATACAAGCCAACCACGGCCTCCAGAAGAAGATGTTGGCGACCTCGTATTGGGAATCCCCGAACATCGCCTCGCTCCA
GTCAATGACCGCTGTTATGCGGCCATTGTCCGTCAGGACATTGTTGGAGCCGAAATCCGCGTGCACGAGGTGCCGGACTTCGGGGCAGT
CCTCGGCCCAAAGCATCAGCTCATCGAGAGCCTGCGCGACGGACGCACTGACGGTGTCGTCCATCACAGTTTGCCAGTGATACACATGG
GGATCAGCAATCGCGCATATGAAATCACGCCATGTAGTGTATTGACCGATTCCTTGCGGTCCGAATGGGCCGAACCCGCTCGTCTGGCT
AAGATCGGCCGCAGCGATCGCATCCATAGCCTCCGCGACCGGCTGAAGAACAGCGGGCAGTTCGGTTTCAGGCAGGTCTTGCAACGTGA
CACCCTGTGCACGGCGGGAGATGCAATAGGTCAGGCTCTCGCTGAACTCCCCAATGTCAAGCACTTCCGGAATCGGGAGCGCGGCCGAT
GCAAAGTGCCGATAAACATAACGATCTTTGTAGAAACCATCGGCGCAGCTATTTACCCGCAGGACATATCCACGCCCTCCTACATCGAA
GCTGAAAGCACGAGATTCTTCGCCCTCCGAGAGCTGCATCAGGTCGGAGACGCTGTCGAACTTTTCGATCAGAAACTTCTCGACAGACG
TCGCGGTGAGTTCAGGCTTTTTCATAGGGATCAGCTTGGGCTGTCCTCTCCAAATGAAATGAACTTCCTTATATAGAGGAAGGGTCTTG
CGAAGGATAGTGGGATTGTGCGTCATCCCTTACGTCAGTGGAGATGTCACATCAATCCACTTGCTTTGAAGACGTGGTTGGAACGTCTT
CTTTTTCCACGATGCTCCTCGTGGGTGGGGGTCCATCTTTGGGACCACTGTCGGCAGAGGCATCTTGAATGATAGCCTTTCCTTTATCG
CAATGATGGCATTTGTAGGAGCCACCTTCCTTTTCTACTGTCCTTTCGATGAAGTGACAGATAGCTGGGCAATGGAATCCGAGGAGGTT
TCCCGAAATTACCCTTTGTTGAAAAGTCTCAATAGCCCTTTGGTCTTCTGAGACTGTATCTTTGACATTTTTGGAGTAGGGGTACGATA
ACATTAACGTTTACAATTTCGCGCCATTCGCCATTCAGGCTGCGCAACTGTTGGGAAGGGCGATCGGTGCGGGCCTCTTCGCTATTACG
CCAGCTGGCGAAAGGGGGATGTGCTGCAAGGCGATTAAGTTGGGTAACGCCAGGGTTTTCCCAGTCACGACGTTGTAAAACGACGGCCA
GTGAATTGTAATACGACTCACTATAGGGCGAATTGGGTACCGTTTAAACAAGCTTCCTAGCCCTGCAGGGGATCCTGGGTTTTGTATCT
AAAGATCAAAGGAATAACATTTTTGGGAGATGAGAAACTTCAAAAGATGCGATTTAGTTTTTGTGGAAAAGTGGGGAATTTAATTCGAT
TGACGTTAGGAACTTTTTCTCTATGGATTCTTTGGGTTTGTGATTAATGGCATTGAAGGTACAAAGGAAAAGGTTTGGCCAACAAAATC
AAGAACCACAAAGAGAGAGAGAGAGAGAGGAAACACACAGACAAAACATATGAAAGAATGATTAAGATAACTGCCACAAATATAACTGCAA
AATAACATACTCCTTAAGAAAATTAGTGGGGCCTTCTTTGTTATGGAAGAATGTGCAAATCTTTTTATATGGTTAAGTATATTAGTATT
ATTATATCAAAATTCATTTTTGACTATATATAGGTGATTGTGTCGAAATTCACGTAAGATCTTTGTAACATTTTTTCACACTAGTTAAT
TTTACATGTATTATAGATTCGCTGCAAAAAAAAAAAAAAAAAAAAAAAGAAAATAGAAAAATTTCTGTTTTTTCACACTAGGCCGATTT
GATTGATCAAAACCGATTTTAAAAGCTTAAACTAATAAATCGATTTCATTCACCAATTTATTAAAATTAGATCGTTTTGGAGGTTCAAG
AATATCTGGTCGCTGTTTAAGACGATATTTTGAATCTTGAACCGTTGAAACTATATATACTAGGTATATTTACTGTATGTTGTGAAAGG
GTTTACTATCACATGGCTATTATATAAGACCGTACTAACATATGCAAATATTATATGTGGATCAAAGTACCACAAATTTTGAGTGAACC
AAAAACAAAAGGTTAGAGATAGAAAAATATGCATGTAAAAAATAATAAGAGATGAAAGAAATATTAATGGGAGATGGGTGGGAAATGAA
TAGATTGGTGTCTTCGTCACTTTTACTTTTTTTTTTTTTTATTGTTTGTTTGATAGTACCAAGAGATTCTCTATTCTATTCTTTCGTGA
TGGTTTATTTTAGGGTATATTACGTGACCTGTAGTTCATCCTCTCCAACTCTCAAACCCACAAATTATTCACTATACATATATCCAAAG
ATTATTTATTCAAAGATATGACTAAAAATCTCTATACACGTGTGTATGTAATAGAAAGGTGCAAGGATTTTGGTGTGGCACCACCTTCA
AGACTTTTTTTTTATCAAGACCTAACTCTTACCACTTTGAAAGCAGCTTATCTTATTTCTTTAGATAAAACAATTGATAAGCGAGTGCT
TTAAACTTCACAAAACTACAATCTACTTTTTATTCGATAAAACAAAAGATTTTATTAGTCTTTTTCATTAGGGTCCCAAATGATTAATA
TGTTCACGATCACGAAATTTCTTCTTGCGTCCCGTAAGCATTTATATTAATCAAACAAAAAGGAAATTTGTTGCTTCCTACAATGGGAA
GTGGGAACCTTTGCCATCTTCATAAATGTTTTAAATCTTTTTTTTTTCTTTCTAATGTGTGTTTCATAAACACCAGCGACTTTATTGAA
AAGAGGAACACGTAAATTGTATTCTCAAAGAACAATTTTTTTAGGAAGAGTAACATTTGTTTTCCTTATCTTAATATAAGCAACGAAAT
CAATCGTGCATATAAAAGTGAAATGCTATTTTGAATTTTCTAAACTTGACTAGCAAATTTTCACTAAAAAATAAAATTAAAAAGTAACG
ATAAAGTAGTTGTCGTATAGTCTACTCCACATGAATAAAATAATAAAGTAAATCAATAGAATGTAAATCTCTTAATTAATCATACTTAT
TATATAATCACAGTGGCAAAGTGGAATAAAAAAAAAAAGAGGAGAATTGGATCCCGTCGGATTCTATATTTAAAGATCGCTAAACATCTC
AAATCTGCCACACGTGGTCATACTTAATAGCAGCCATTAACGAAGTAATAAATTAGTCGATAATAATGTTTAACCAACATCGTATTTCT
AGATCCATGAATTAGTTGGAGTAATTAACATCATTGTTTCTTAATTACGTTTAACAAGCATAGAGAAAACAATATTGTCACATATGTTA
GTATAAGTTATCATAATCATTTATAAAAGCTTGTTATTAATTTTTTATATTGAAATTACTACTTTGTGTCTAATCGGAAAATGATGGAG
TACTAAAATTTGAAGGACTAAAATACGTCATTATTGTTAGAGAAAACAATTACGCATAAGTGTGATTTTTTGGAAGTGGAAAAGTTAGT
ATGTCCCTCTATATGATATGATGGAAGTTGTTGTTCTTTTTGTGTGGATAAACATCATTTTTCCGTGACGTAATTTTTAATTTCAGTAT
TCATTTTATTATTCTCACTTCTTATTATCCCTTTTATATTCGCATAATTGTTTCTAAGGATTTCTATTCCCTTTTTGTTATTTTATAGG
TACACTATCTTATGGATGGTGTCAACAAGCATGTACACTATCTTCCCACTACCACATATTGTCACAGAATGTTTGGTACGCGTGCTTCT
ATATACTTTTAATAGTACATAACAACAACAAATGAATTATGTAAGACAATTTGTTAAACTGATGTCAAAAACGGAAATACTACAAAAGA
CAGTATAATTGAATTACGGGTTTAATGTGTTGACAAAATATAATAAATGTAGCTCATAAAACTTCAATAATTCACTTAAATAAATTCTA
ATGAACCTCATAACGAATTAGATATTTTAACTAATCTTATCATGGTTTTACGAATTAATTATAATTTTTTTTTTTTATTCTTTGAACAAT
TGAACTAAACAATATTAAACCGGACTTTAAGTTAAAACTACTAAAAACATTATTAGTTTATCAATAGAAATGATCATAGCAATTGTATA
CATTATAATGTGTAATTTTAAAATCCTAAAGCATATATAGTATATATCCCTTTGATTTTCATAGAAAAAAAGTAGAAAAAAATAACTAT
TGGGCCGATCTATAACTATTGGGCCGGTGGGTCCAACAACTAAGAACAGTAGATATTGTGTCGATTGAAGTTTTGAGATGCAAAACGTG
TGCTGTTTTGAGAACACAAAAACAGCATAAACAAACGAGACCCGCAACTATAACATTCCGTAAATACCCTCATTCTCCCATCATATTTT
CAAAAACTACCCTTCCCCCTATTTCCATGGCCCTTCTCTCAGTTTTCCCGCGAGAAATCCTCTCCTTCTTCCATAATCATCAAAAACAA
AATGCAAAAACCCAAAAAAACAAAAAATCACACGACGCAACCACCTAGCCTCTCTGTCCATCAATCTCAATCTCTCTCTGTGTTAATGC
GGAATCCGATTTAACACCGGAATCCGCCATGGAAGGGAGGCGGTGTCGAGGTGCAGTAGTCCACCTTCACTTGCTCTCTTTTTTCCGAA
GAATCTGGTTCGTTTCTCTTTGAATTATCTCTTTCTCTGTTCTTCTTCTTTTTCTAAAGTCTCGAATATATCTTCATTATCATTACTTT
ATTTGCTTCGTTCTTCCGCAGTTTTGTGCAAAGTTTTAACGATTTTTTGGTTTTGAACGAAGGCACAGAGAGAAGGCGCGTCGAC*ATGG*
*ACTACAAAGACGATGACGACAAG*GTCGACGGCGCGCCCGGCGGAATGATGATGCAGAGAAACTGTTTCGGATTTAACCTAAAGAACCGA

GGAGGTGAAAAGAAGAAAGCATCCAAATCTTTCAGGGAAGGAGTTAAAAAAATCAGATCAGAAGGTCTAATAACCATCGGTAAATCCGT
TACAAGAGCGGTTTTTCCAGAAGACTTAAGAATAACAGAGAAGAAAATCTTTGATCCTCAAGACAAAACACTTCTCGTATGGAACAGAT
TGTTCGTCATTTCGTGTATTTTAGCTGTCTCTGTTGATCCTTTGTTCTTCTATCTTCCCATTGTCGATAATTCCGGAAGCAGCTGTATA
GGGATTGATACGAAACTAGCTGTTACAACGACGACTCTTAGAACCATTGTTGACGTTTTTTACTTAACTAGAATGGCTCTTCAGTTTCG
TACAGCTTATATAGCTCCTTCTTCTCGTGTGTTTGGTCGTGGTGAGCTTGTGATTGATCCTGCAAAGATCGCTGAACGCTATTTAACCC
GTTATTTCGTCGTCGATTTTCTTGCAGTGCTGCCTTTACCGCAGGTAAAACAGAGTGAAAAGATCCTTTGATGGTTACTAAAAATATTG
GACTTTTGGGATTGATTATGATGTTTTGTGTGGATATTACAGATTGCAGTGTGGAAGTTTCTTCACGGGTCTAAAGGATCGGATGTGTT
ACCAACGAAAACGGCATTGTTGAACATTGTAATTGTGCAGTATATTCCGAGATTCGTGAGGTTTATTCCGTTAACATCAGAGTTGAAGA
AAACTGCAGGAGCTTTTGCCGAAGGAGCTTGGGCTGGTGCTGCTTATTATCTTCTCTGGTATATGCTTGCAAGCCACGTAAGCGTTTCT
ATGTATATATGACTCTTCTTAATTAGTGTTTTGAGACATTTATTCATCATTGGTCACTTTCTGACTTTACAAACACATTTTGACAGATA
ACTGGAGCGTTTTGGTACATGTTGTCAGTGGAGCGTAACGATACTTGTTGGAGATTTGCGTGTAAAGTTCAGCCAGATCCGCGACTCTG
TGTTCAGATTCTGTATTGTGGGACTAAATTTGTGAGCAGTGGAGAAACTGAATGGATCAAAACAGTCCCTGAGCTTCTTAAGAGCAATT
GTTCTGCTAAAGCAGATGATTCAAAGTTTAACTATGGGATATACGGTCAGGCTATATCTTCAGGCATTGTATCATCAACAACATTTTTC
TCCAAGTTCTGTTATTGTCTATGGTGGGGTCTTCAAAATCTCAGGTAAGGCGGGGGCAACTCATTGTCATTGGTTCAACCGATAGCTTG
TGCAGAGTACTCTTGACATCTTTGGTGTTTTCATGAACAGCACGTTAGGTCAAGGGCTGCAGACAAGTACATTTCCAGGAGAGGTTTTG
TTTTCCATTGCGATTGCTATAGCTGGGCTTCTTCTTTTCGCGCTTCTCATCGGGAATATGCAGACTTATCTTCAGTCACTTACTGTTCG
TCTCGAGGAAATGAGGATCAAAAGACGTGACTCAGAACAGTGGATGCATCACAGGTCACTTCCACAAAATTTGAGGGAAAGAGTCAGAC
GCTATGATCAATACAAATGGTTAGAGACAAGAGGTGTAGACGAAGAAAACATAGTTCAGAGTTTACCTAAGGATCTCAGAAGAGACATC
AAACGCCATCTCTGTCTGAATTTAGTTAGGAGGGTAAGTTTTCACAATCAGAATGCTAAAATTGCTTGAGATACTAACTAACTATGTGT
TCTGTTTCTACAGGTTCCTCTGTTTGCTAATATGGATGAAAGATTACTTGACGCAATCTGTGAGAGATTAAAGCCGAGTCTTTTCACGG
AAAGCACTTACATTGTGCGTGAAGGAGACCCCGTTAACGAAATGATGTTCATAATCCGAGGCCGGTTAGAAAGTGTAACAACAGATGGG
GGAAGAAGCGGTTTCTTCAACAGAGGGTTGTTAAAAGAAGGAGACTTTTGTGGTGAAGAGCTTCTGACATGGGCTCTTGACCCTAAAGC
AGGCTCAAACTTACCTTCTTCCACAAGAACAGTGAAGGCTCTGACTGAAGTAGAGGCTTTCGCTCTTGAAGCTGAAGAGCTGAAGTTTG
TAGCAAGTCAGTTTAGGAGACTTCATAGTCGACAGGTTCAACAAACATTTAGGTTTTACTCTCAGCAATGGAGAACTTGGGCTTCTTGC
TTCATCCAAGCTGCTTGGCGTCGCTATTCAAGAAGGAAAAACGCTGAATTGAGACGGATTGAAGAAAAAGAAGAAGAATTGGGTTATGA
AGATGAATATGATGATGAGAGTGACAAGAGGCCTATGGTTATCACAAGAAGTGAGTCTTCTTCTAGACTGCGTTCAACGATATTTGCAT
CAAGATTTGCTGCTAATGCATTGAAAGGGCACAGACTTAGGAGCTCAGAGAGTTCAAAGACCTTGATAAATCTGCAAAAGCCTCCGGAA
CCTGATTTTGATGCTGAATGAGGCCTAGGCGCGGTCGCGGGGATCACTAGTGAATTCGCGGCCGCGGGGCCCGGCTGGAGCCACCCGCA
GTTCGAAAAATGAGAGCTCGAATTTCCCCGATCGTTCAAACATTTGGCAATAAAGTTTCTTAAGATTGAATCCTGTTGCCGGTCTTGCG
ATGATTATCATATAATTTCTGTTGAATTACGTTAAGCATGTAATAATTAACATGTAATGCATGACGTTATTTATGAGATGGGTTTTTAT
GATTAGAGTCCCGCAATTATACATTTAATACGCGATAGAAAACAAAATATAGCGCGCAAACTAGGATAAATTATCGCGCGCGGTGTCAT
CTATGTTACTAGATCGGGAATTCAGCTTTTGTTCCCTTTAGTGAGGGTTAATTCCGAGCTTGGCGTAATCATGGTCATAGCTGTTTCCT
GTGTGAAATTGTTATCCGCTCACAATTCCACACAACATACGAGCCGGAAGHCATAAAGTGTAAAGCCTGGGGTGCCTAATGAGTGAGCT
AACTCACATTAATTGCGTTGCGCTCACTGCCCGCTTTCCAGTCGGGAAACCTGTCGTGCCAGCTGCATTAATGAATCGGCCAACGCGCG
GGGAGAGGCGGTTTGCGTATTGGGCGCTCTTCCGCTTCCTCGCTCACTGACTCGCTGCGCTCGGTCGTTCGGCTGCGGCGAGCGGTATC
AGCTCACTCAAAGGCGGTAATACGGTTATCCACAGAATCAGGGGATAACGCAGGAAAGAACATGAAGGCCTTGACAGGATATATTGGCG
GGTAAACTAAGTCGCTGTATGTGTTTGTTTGAGATCTCATGTGAGCAAAAGGCCAGCAAAAGGCCAGGAACCGTAAAAAGGCCGCGTTG
CTGGCGTTTTTCCATAGGCTCCGCCCCCCTGACGAGCATCACAAAAATCGACGCTCAAGTCAGAGGTGGCGAAACCCGACAGGACTATA
AAGATACCAGGCGTTTCCCCCTGGAAGCTCCCTCGTGCGCTCTCCTGTTCCGACCCTGCCGCTTACCGGATACCTGTCCGCCTTTCTCC
CTTCGGGAAGCGTGGCGCTTTCTCATAGCTCACGCTGTAGGTATCTCAGTTCGGTGTAGGTCGTTCGCTCCAAGCTGGGCTGTGTGCAC
GAACCCCCCGTTCAGCCCGACCGCTGCGCCTTATCCGGTAACTATCGTCTTGAGTCCAACCCGGTAAGACACGACTTATCGCCACTGGC
AGCAGCCACTGGTAACAGGATTAGCAGAGCGAGGTATGTAGGCGGTGCTACAGAGTTCTTGAAGTGGTGGCCTAACTACGGCTACACTA
GAAGAACAGTATTTGGTATCTGCGCTCTGCTGAAGCCAGTTACCTTCGGAAGAAGAGTTGGTAGCTCTTGATCCGGCAAACAAACCACC
GCTGGTAGCGGTGGTTTTTTTGTTTGCAAGCAGCAGATTACGCGCAGAAAAAAAGGATCTCAAGAAGATCCTTTGATCTTTTCTACGGG
GTCTGACGCTCAGTGGAACGAAAACTCACGTTAAGGGATTTTGGTCATGAGATTATCAAAAAGGATCTTCACCTAGATCCTTTTAAATT
AAAAATGAAGTTTTAAATCAATCTAAAGTATATATGTGTAACATTGGTCTAGTGATTAGAAAAACTCATCGAGCATCAAATGAAACTGC
AATTTATTCATATCAGGATTATCAATACCATATTTTTGAAAAAGCCGTTTCTGTAATGAAGGAGAAAACTCACCGAGGCAGTTCCATAG
GATGGCAAGATCCTGGTATCGGTCTGCGATTCCGACTCGTCCAACATCAATACAACCTATTAATTTCCCCTCGTCAAAAATAAGGTTAT
CAAGTGAGAAATCACCATGAGTGACGACTGAATCCGGTGAGAATGGCAAAAGTTTATGCATTTCTTTCCAGACTTGTTCAACAGGCCAG
CCATTACGCTCGTCATCAAAATCACTCGCATCAACCAAACCGTTATTCATTCGTGATTGCGCCTGAGCGAGACGAAATACGCGATCGCT
GTTAAAAGGACAATTACAAACAGGAATCGAATGCAACCGGCGCAGGAACACTGCCAGCGCATCAACAATATTTTCACCTGAATCAGGAT
ATTCTTCTAATACCTGGAATGCTGTTTTCCCTGGGATCGCAGTGGTGAGTAACCATGCATCATCAGGAGTACGGATAAAATGCTTGATG
GTCGGAAGAGGCATAAATTCCGTCAGCCAGTTTAGTCTGACCATCTCATCTGTAACAACATTGGCAACGCTACCTTTGCCATGTTTCAG
AAACAACTCTGGCGCATCGGGCTTCCCATACAATCGGTAGATTGTCGCACCTGATTGCCCGACATTATCGCGAGCCCATTTATACCCAT
ATAAATCAGCATCCATGTTGGAATTTAATCGCGGCCTTGAGCAAGACGTTTCCCGTTGAATATGGCTCATAACACCCCTTGTATTACTG
TTTATGTAAGCAGACAGTTTTATTGTTCATGATGATATATTTTTATCTTGTGCAATGTAACATCAGAGATTTTGAGACACAACGTGGCT
TTGTTGAATAAATCGAACTTTTGCTGAGTTGAAGGATCAGATCACGCATCTTCCCGACAACGCAGACCGTTCCGTGGCAAAGCAAAAGT
TCAAAATCACCAACTGGTCCACCTACAACAAAGCTCTCATCAACCGTGGCTCCCTCACTTTCTGGCTGGATGATGGGGCGATTCAGGCG
ATCCCCATCCAACAGCCCGCCGTCGAGCGGGCT
